# Effect of Front-of-Package Information, Fruit Imagery, and High–Added Sugar Warning Labels on Parent Beverage Choices for Children: A Randomized Clinical Trial

**DOI:** 10.1001/jamanetworkopen.2022.36384

**Published:** 2022-10-13

**Authors:** Aviva A. Musicus, Christina A. Roberto, Alyssa J. Moran, Sarah Sorscher, Eva Greenthal, Eric B. Rimm

**Affiliations:** 1 Department of Social and Behavioral Sciences, Harvard T.H. Chan School of Public Health, Boston, Massachusetts; 2 Department of Medical Ethics and Health Policy, Perelman School of Medicine at the University of Pennsylvania, Philadelphia; 3 Department of Health Policy and Management, Johns Hopkins Bloomberg School of Public Health, Baltimore, Maryland; 4 Center for Science in the Public Interest, Washington, District of Columbia; 5 Department of Nutrition, Harvard T.H. Chan School of Public Health, Boston, Massachusetts

## Abstract

**Importance:**

Fruit drinks are widely consumed by young children, and many parents mistakenly believe that these drinks are healthy, potentially due to front-of-package claims and imagery. Research is needed on the influence of this marketing and how labeling regulations could change behavior.

**Objective:**

To assess the effects of a front-of-package 100% vitamin C claim, fruit imagery, percentage juice and teaspoons of added sugar disclosures, and high–added sugar warnings on parents’ choices, knowledge, and perceptions of beverages.

**Design, Setting, and Participants:**

This randomized clinical trial was conducted May to July 2021 as a single-exposure (no follow-up) online survey of primary caregivers of children ages 0 to 5 years throughout the US.

**Interventions:**

Participants were shown no–, low–, and high–added sugar beverages and asked to choose 1 for their child. Participants were randomized to see high–added sugar beverages with 1 of 7 front-of-package conditions: (1) claim and imagery (control); (2) no claim; (3) no imagery; (4) no claim or imagery; (5) claim, imagery, and percentage juice disclosure; (6) claim, imagery, and warning; or (7) claim, imagery, warning, and teaspoons of added sugar disclosure.

**Main Outcomes and Measures:**

Primary outcomes were type of beverage chosen (eg, high–added sugar beverage) and resulting calories and added sugar (in grams). Secondary outcomes were fruit drink knowledge (added sugar and percent juice) and perceptions.

**Results:**

There were 5005 participants included in the final analysis (mean [SD] age, 31.5 [8.3] years; 3587 female participants [71.7%]), including 714 participants in group 1, 717 participants in group 2, 710 participants in group 3, 717 participants in group 4, 708 participants in group 5, 729 participants in group 6, and 710 participants in group 7. Compared with participants in the control group, who had a mean (standard error [SE]) of 9.4 (0.5) g of added sugar and 81.9 (1.6) kcal in chosen beverages, only participants who saw warnings with teaspoons of added sugar disclosures had significantly reduced added sugar (−1.3 g; 95% CI, −2.6 to −0.1 g [−14.2%; 95% CI, −26.7% to −1.8%]; *P* = .04) and calories (−5.3 kcal; 95% CI, −9.8 to −0.9 kcal [−6.5%; 95% CI, −11.8% to −1.3%]; *P* = .02) in selected beverages. In warning conditions (ie, 6 and 7) compared with the control group (mean [SE] 41.0% [1.8%]), the proportion of participants choosing high–added sugar beverages was significantly reduced, by 5.5 percentage points (95% CI, 0.5 to 10.5 percentage points [13.4%; 95% CI, 1.2% to 25.6%]; *P* = .03) and 6.4 percentage points (95% CI, 1.4 to 11.4 percentage points [15.6%; 95% CI, 3.3% to 27.8%]; P = .01), respectively. The no claim or imagery condition (4) significantly reduced the proportion of parents choosing high–added sugar beverages (−7.6 percentage points; 95% CI, −12.6 to −2.6 percentage points [−18.4%; 95% CI, −30.6% to −6.3%]; *P* = .003). Percentage juice disclosures did not affect beverage choice.

**Conclusions and Relevance:**

These findings suggest that added sugar warnings and prohibitions of front-of-package claims and imagery may reduce parents’ purchases of high–added sugar beverages for their young children but that percentage juice disclosures may not change behavior.

**Trial Registration:**

ClinicalTrials.gov Identifier: NCT04811690

## Key Points

**Question:**

What are the effects of a front-of-package 100% vitamin C claim, fruit imagery, percentage juice and teaspoons of added sugar disclosures, and high–added sugar warnings on parents’ beverage choices for their children?

**Findings:**

In this randomized clinical trial of 5005 parents of children aged 0 to 5 years, 15.6% fewer parents chose a high–added sugar beverage when packages displayed warnings with teaspoons of added sugar disclosures, reducing added sugar and calories in chosen beverages by 14.2% and 6.5%, respectively; 18.4% fewer chose a high–added sugar beverage when shown packages without a 100% vitamin C claim or fruit imagery.

**Meaning:**

These findings suggest that adding warnings or removing claims and imagery from high–added sugar beverages may reduce parents’ purchases of these beverages for their children.

## Teaser

This randomized clinical trial investigates the effect of vitamin C claims, fruit imagery, percentage juice and added sugar disclosures, and sugar warnings on parents’ choices of beverages for children.

## Introduction

Most young children in the US consume sugar-sweetened beverages (SSBs),^1,2,3^ predisposing these children to tooth decay, obesity, type 2 diabetes, and other chronic diseases.^4,5,6^ The most frequently consumed SSBs among children aged 1 to 8 years are fruit drinks (ie, fruit-flavored drinks containing <100% juice), which account for 20% of added sugars consumed by children aged 1 year^3^ and 11% of added sugars consumed by children aged 2 to 8 years.^7,8^ Reducing fruit drink consumption among young children could thus help prevent chronic diseases later in life.

One potential driver of fruit drink consumption is the use of misleading front-of-package (FOP) claims and imagery. Nutrient content claims (eg, 100% vitamin C) and fruit imagery are prevalent on fruit drink packages^9,10,11^ but are not reliable indicators of product healthfulness or juice content.^9,11,12^ Research has shown that this marketing misleads consumers.^13,14,15,16,17,18^ In a 2022 study,^18^ parents who viewed fruit drinks with claims were more likely to incorrectly believe that these products did not contain added sugar or were 100% juice. Correcting these misperceptions could enhance parents’ knowledge and encourage healthier beverage purchases.

There are multiple regulatory options to address misleading FOP marketing, but effects of such options alone and in combination have not been well-studied. Some options include restricting nutrient content claims or fruit imagery on products with high levels of added sugar, requiring FOP percentage juice content disclosures^19^ (in addition to currently mandated back-of-pack disclosures^20^), or requiring added-sugar warnings if these added sugars exceed a predetermined level. Evidence suggests that added-sugar warnings may reduce SSB purchases,^21,22,23,24,25^ and the US Congress has introduced a bill to mandate such warnings.^26^ Combining warnings with quantitative added-sugar disclosures (eg, teaspoons of added sugar/serving) may further discourage SSB consumption.^27,28,29^

Prior consumer research on FOP marketing has largely been conducted with unbranded or mock beverages, so there are limited data on the impact of FOP changes in actual products in a realistic retail setting, in which FOP claims, imagery, disclosures, warnings, and branding interact to affect behavior.^13,30^ The goal of this study was to compare independent and combined effects of common FOP marketing tactics (ie, 100% vitamin C claims and fruit imagery), nutrition disclosures (ie, teaspoons of added sugar/serving and percentage juice content), and high–added sugar warnings on parents’ beverage selections for their young children and on parents’ knowledge and perceptions of fruit drinks with varied levels of added sugar.

## Methods

This randomized clinical trial followed the Consolidated Standards of Reporting Trials (CONSORT) reporting guideline for randomized clinical trials. The Harvard T.H. Chan School of Public Health Institutional Review Board approved this study, and all participants gave electronic informed consent. The trial protocol appears in Supplement 1.

### Participants

**Figure 1.**
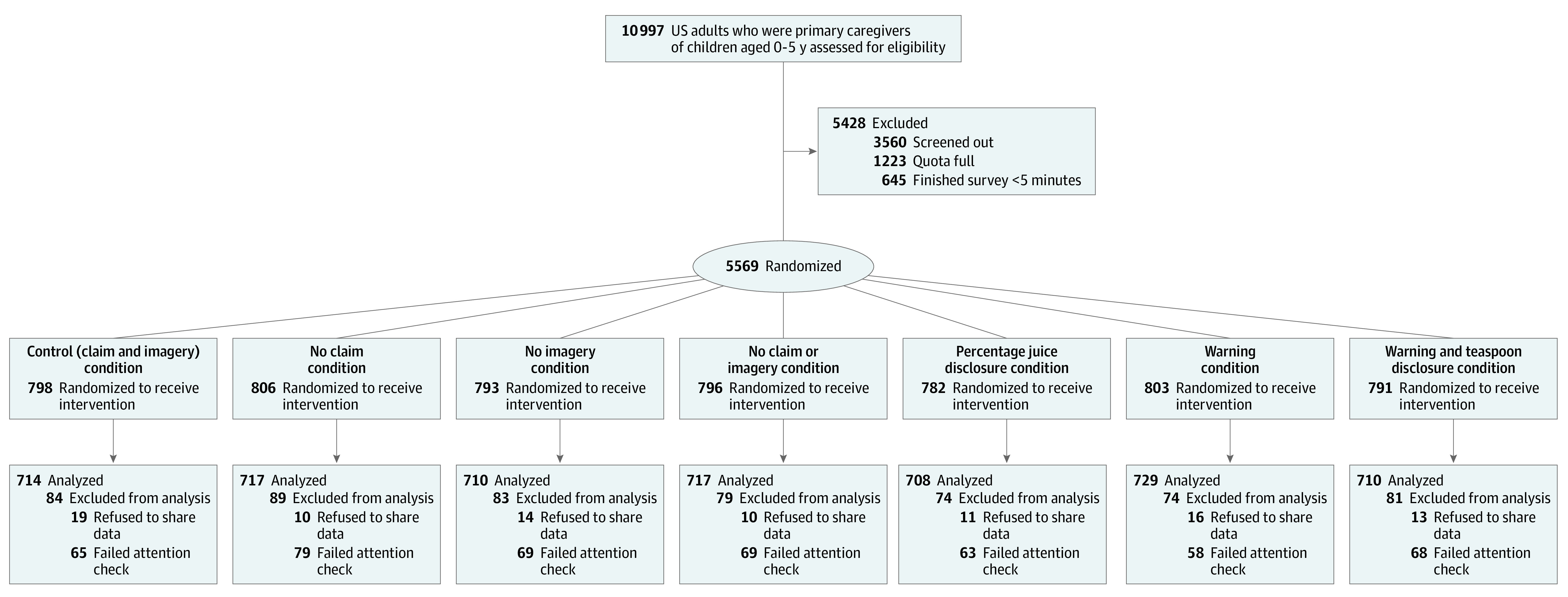
Study Flow Chart

Participants were recruited for an online randomized clinical trial (single exposure, no follow-up) from May to July 2021 through Qualtrics, a survey-sampling and administration company that recruits participants from a network of research panels via online ads, promotions, social networks, and online and mobile games. Participants were sampled to reflect the US educational distribution based on 2010 Census data, with oversampling for Black and African American and Hispanic participants because Black and Hispanic children have the highest national fruit drink consumption rates.^31,32^ Participants self-reported race and ethnicity using investigator-defined options. Race and ethnicity options were Asian; Black or African American; Latinx/o/a or Hispanic; Native American; Native Hawaiian, Pacific Islander, or Alaska Native; White, and other. Other represents anyone who self-identified as other or as more than 1 category (excluding Hispanic). The Hispanic category included anyone who indicated they were Hispanic or Hispanic and 1 or more other races. For example, if someone said they were White and Hispanic, that was counted toward White and Hispanic separately, but if they said they were White, Asian, and Hispanic, that was counted toward Hispanic and other separately. Participants had to be living in the US, aged 18 years or older, and primary caregivers of at least 1 child aged 0 to 5 years. We excluded participants for completing the survey in less than 5 minutes (one-third of the median completion time of 15 minutes), failing an attention check (multiple choice question asking, “What month is it?”), or refusing to share their data. There were no missing data. The final sample had 5005 participants based on a priori sample size calculations informed by previous literature^24,33,34^ (Table 1 and Figure 1).

**Table 1.** Participant Characteristics

Characteristic^a^	Participants, No. (%)							
	Total (N = 5005)	Control (claim and imagery) (n = 714)	No claim (imagery only) (n = 717)	No imagery (claim only) (n = 710)	No claim or imagery (n = 717)	% Juice disclosure (n = 708)	Warning (n = 729)	Warning and teaspoon disclosure (n = 710)
Gender								
Female	3587 (71.7)	500 (70.0)	517 (72.1)	508 (71.6)	535 (74.6)	499 (70.5)	515 (70.6)	513 (72.3)
Male	1363 (27.2)	207 (29.0)	193 (26.9)	191 (26.9)	172 (24.0)	199 (28.1)	207 (28.4)	194 (27.3)
Other	55 (1.1)	7 (1.0)	7 (1.0)	11 (1.5)	10 (1.4)	10 (1.4)	7 (1.0)	3 (0.4)
Age, mean (SD), y	31.5 (8.3)	31.5 (8.4)	31.4 (8.6)	31.6 (8.8)	31.3 (8.1)	31.5 (8.1)	31.4 (8.0)	31.4 (7.9)
BMI, mean (SD)	27.6 (8.4)	27.6 (7.4)	27.6 (7.9)	27.3 (6.8)	27.3 (7.2)	27.7 (7.5)	28.0 (13.0)	27.5 (7.3)
Diagnosed health condition								
Prediabetes	169 (3.4)	26 (3.6)	23 (3.2)	24 (3.4)	15 (2.1)	21 (3.0)	33 (4.5)	27 (3.8)
Type 2 diabetes	264 (5.3)	35 (4.9)	45 (6.3)	34 (4.8)	30 (4.2)	41 (5.8)	45 (6.2)	34 (4.8)
Obesity	435 (8.7)	57 (8.0)	76 (10.6)	56 (7.9)	59 (8.2)	62 (8.8)	66 (9.1)	59 (8.3)
Race and ethnicity^b^								
Asian	185 (3.7)	25 (3.5)	30 (4.2)	29 (4.1)	24 (3.4)	24 (3.4)	27 (3.7)	26 (3.7)
Black or African American	1154 (23.1)	150 (21.0)	173 (24.1)	153 (21.6)	173 (24.1)	160 (22.6)	171 (23.5)	174 (24.5)
Hispanic or Latinx/o/a	1126 (22.5)	160 (22.4)	163 (22.7)	162 (22.8)	180 (25.1)	150 (21.2)	156 (21.4)	155 (21.8)
Native American	78 (1.6)	7 (1.0)	7 (1.0)	14 (2.0)	12 (1.7)	11 (1.6)	12 (1.7)	15 (2.1)
Native Hawaiian, Pacific Islander, or Alaska Native	30 (0.6)	10 (1.4)	4 (0.6)	2 (0.3)	1 (0.1)	4 (0.6)	4 (0.6)	5 (0.7)
White	2475 (49.5)	363 (50.8)	344 (48.0)	364 (51.3)	337 (47.0)	358 (50.6)	367 (50.3)	342 (48.2)
Other^c^	305 (6.1)	46 (6.4)	44 (6.1)	36 (5.1)	38 (5.3)	45 (6.4)	44 (6.0)	52 (7.3)
Education								
≤High school degree	1631 (32.6)	246 (34.5)	231 (32.2)	237 (33.4)	245 (34.2)	231 (32.6)	226 (31.0)	215 (30.3)
Associate degree or some college	1696 (33.9)	241 (33.8)	252 (35.2)	230 (32.4)	227 (31.7)	259 (36.6)	249 (34.2)	238 (33.5)
College or graduate degree	1678 (33.5)	227 (31.8)	234 (32.6)	243 (34.2)	245 (34.2)	218 (30.8)	254 (34.8)	257 (36.2)
Annual household income, $								
<25 000	1150 (23.0)	154 (21.6)	178 (24.8)	163 (23.0)	155 (21.6)	193 (27.3)	156 (21.4)	151 (21.3)
25 000-49 999	1254 (25.1)	204 (28.6)	158 (22.0)	193 (27.2)	180 (25.1)	177 (25.0)	173 (23.7)	169 (23.8)
50 000-74 999	895 (17.9)	135 (18.9)	130 (18.1)	127 (17.9)	135 (18.8)	106 (15.0)	132 (18.1)	130 (18.3)
75 000-99 999	646 (12.9)	70 (9.8)	94 (13.1)	88 (12.4)	97 (13.5)	94 (13.3)	98 (13.4)	105 (14.8)
100 000-124 999	442 (8.8)	64 (9.0)	67 (9.3)	56 (7.9)	64 (8.9)	61 (8.6)	71 (9.7)	59 (8.3)
125 000-149 999	284 (5.7)	39 (5.5)	40 (5.6)	35 (4.9)	33 (4.6)	41 (5.8)	53 (7.3)	43 (6.1)
≥150 000	334 (6.7)	48 (6.7)	50 (7.0)	48 (6.8)	53 (7.4)	36 (5.1)	46 (6.3)	53 (7.5)
SNAP participation last 12 mo	1850 (37.0)	268 (37.5)	259 (36.1)	265 (37.3)	252 (35.2)	295 (41.7)	274 (37.6)	237 (33.4)
WIC participation last 12 mo	1423 (28.4)	184 (25.8)	202 (28.2)	197 (27.8)	207 (28.9)	228 (32.2)	221 (30.3)	184 (25.9)
Household size, mean (SD), No. of people	4 (1.4)	4 (1.4)	4 (1.4)	3.9 (1.4)	4 (1.5)	4 (1.4)	4 (1.4)	3.9 (1.4)
No. of children, mean (SD)	2 (1.2)	2 (1.2)	2 (1.1)	2 (1.1)	1.9 (1.1)	2.1 (1.2)	2 (1.1)	2 (1.3)
Age of oldest child aged 0-5 y, y								
<1	458 (9.2)	63 (8.8)	68 (9.5)	65 (9.2)	61 (8.5)	57 (8.1)	67 (9.2)	77 (10.9)
1	582 (11.6)	66 (9.2)	91 (12.7)	84 (11.8)	94 (13.1)	78 (11.0)	86 (11.8)	83 (11.7)
2	775 (15.5)	120 (16.8)	117 (16.3)	99 (13.9)	109 (15.2)	113 (16.0)	105 (14.4)	112 (15.8)
3	967 (19.3)	141 (19.8)	135 (18.8)	136 (19.2)	134 (18.7)	131 (18.5)	158 (21.7)	132 (18.6)
4	974 (19.5)	142 (19.9)	133 (18.6)	140 (19.7)	148 (20.6)	137 (19.4)	143 (19.6)	131 (18.5)
5	1249 (25.0)	182 (25.5)	173 (24.1)	186 (26.2)	171 (23.9)	192 (27.1)	170 (23.3)	175 (24.7)
Frequency of child fruit drink consumption								
Never	366 (7.3)	53 (7.4)	56 (7.8)	49 (6.9)	43 (6.0)	48 (6.8)	58 (8.0)	59 (8.3)
≤1 time/mo	512 (10.2)	68 (9.5)	76 (10.6)	68 (9.6)	74 (10.3)	70 (9.9)	81 (11.1)	75 (10.6)
2-3 times/mo	759 (15.2)	113 (15.8)	104 (14.5)	106 (14.9)	94 (13.1)	111 (15.7)	125 (17.2)	106 (14.9)
1-2 times/wk	1014 (20.3)	155 (21.7)	133 (18.6)	150 (21.1)	157 (21.9)	127 (17.9)	142 (19.5)	150 (21.1)
3-6 times/wk	1110 (22.2)	157 (22.0)	156 (21.8)	149 (21.0)	179 (25.0)	151 (21.3)	156 (21.4)	162 (22.8)
Every day	983 (19.6)	133 (18.6)	158 (22.0)	141 (19.9)	141 (19.7)	156 (22.0)	126 (17.3)	128 (18.0)
>1 time/d	261 (5.2)	35 (4.9)	34 (4.7)	47 (6.6)	29 (4.0)	45 (6.4)	41 (5.6)	30 (4.2)

Abbreviations: BMI, body mass index (calculated as weight in kilograms divided by height in meters squared); SNAP, Supplemental Nutrition Assistance Program; WIC, Special Supplemental Nutrition Program for Women, Infants, and Children.

^a^ Full sample characteristics are shown.

^b^ Does not add to 100% because the Hispanic category includes anyone who indicated they were Hispanic or Hispanic and 1 or more race. For example, if someone said they were White and Hispanic, that counted toward White and Hispanic separately, but if they said they were White, Asian, and Hispanic, that counted toward Hispanic and other separately.

^c^ Other includes participants who self-identified as other and anyone who chose more than 1 category, excluding Hispanic.

### Survey Procedure

After providing written informed consent, participants completed an online survey (all questions and image information are in eAppendix 1 and 2 in Supplement 2). In a choice task, participants viewed 12 commonly purchased^11^ multipacks of real beverages in a random order and were instructed to “click on the drink you would like to purchase for your oldest child aged 0-5.” To incentivize realistic shopping behavior, participants were told that at the end of the survey a computer would randomly select whether they would have their selected beverage shipped to them or be given $5. Beverages included 6 high–added sugar beverages (>20% daily value added sugar/serving^35^: 4 fruit drinks, 1 cola, and 1 fruit-flavored soda) and 6 low– or no–added sugar beverages (1 low–added sugar fruit drink, 1 no–added sugar fruit drink, two 100% juices, 1 milk, and 1 water). After participants selected a beverage, an enlarged image appeared and participants were asked to confirm their selection or go back and choose a different beverage.

Participants then answered knowledge and perception questions about 4 fruit drinks (2 high, 1 low, and 1 no added sugar), which they viewed 1 at a time in random order. These drinks had not appeared in the previous choice task but featured the same brands. Participants then reported whether they noticed different package elements and provided demographic information, including age, gender, race and ethnicity, height, weight, chronic disease history, annual household income, household size, number of children aged 0 to 5 years, and Supplemental Nutrition Assistance Program (SNAP) and Special Supplemental Nutrition Program for Women, Infants, and Children (WIC) participation. They also reported the frequency of any fruit drink consumption for their oldest child in that age range.

### Label Randomization

**Figure 2.**
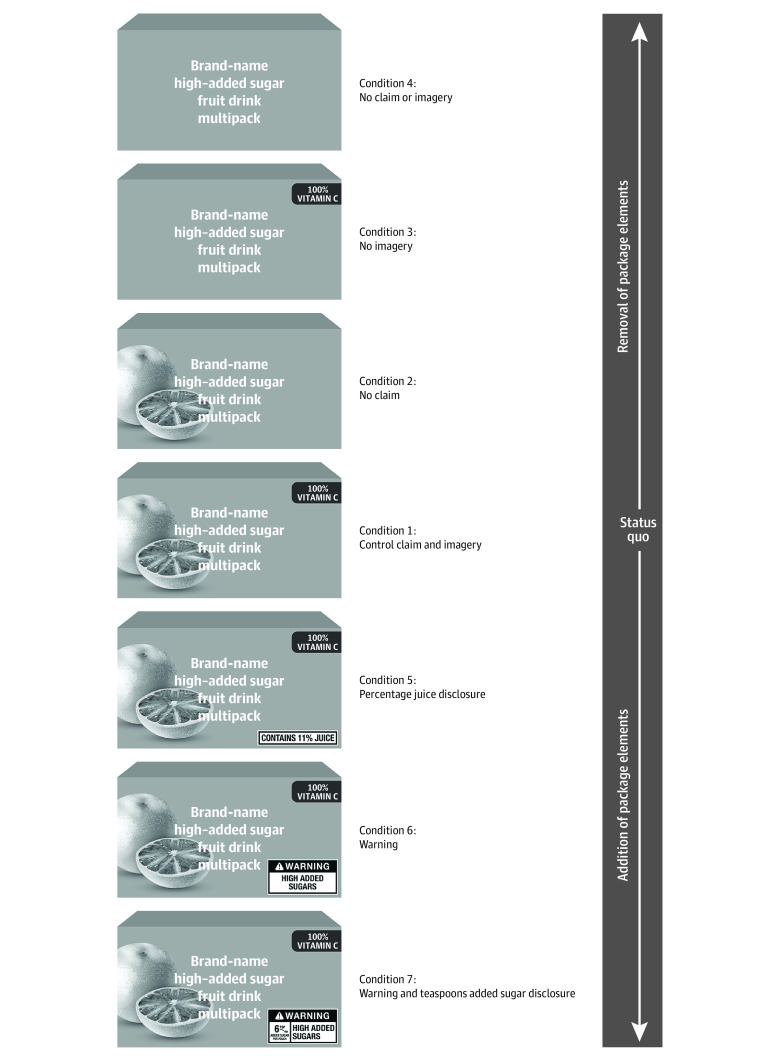
Study Conditions Although images of real branded products were used in this study, product images in Figure 2 are brandless mock-ups for the purposes of publication. The grapefruit images in the figure mock-ups are vector images created by macrovector, designed by Freepik. In the study, a graphic designer modified real product images for each condition to ensure that all packages looked as realistic as possible. For more information about images used in the study, please see eAppendix 2 in Supplement 2.

Participants were randomized (simple randomization) to view high–added sugar beverages throughout the survey with 1 of 7 FOP label designs (Figure 2; eAppendix 2 in Supplement 2). Although images of real branded products were used in this study, product images in Figure 2 are brandless mock-ups for the purposes of publication. The grapefruit images in the figure mock-ups are vector images created by macrovector, designed by Freepik. In the study, a graphic designer modified real beverage package images to make each condition look as realistic as possible. The control condition included high–added sugar beverages with 100% vitamin C claims and fruit imagery because most sweetened drinks for children contain claims and imagery and 100% vitamin C claims are among the most common.^9,10,11,12^ High–added sugar beverages in conditions 2 to 4 lacked 1 or both of these elements (2: no claim; 3: no imagery; 4: no claim or imagery). Conditions 5 to 7 had additional labeling elements (5: claim, imagery, and percentage juice disclosure; 6: claim, imagery, and high–added sugar warning; 7: claim, imagery, high–added sugar warning, and teaspoons of added sugar disclosure). Throughout the survey, 100% vitamin C claims and fruit imagery were shown on all low– and no–added sugar fruit drinks and 100% juices, but these elements were not shown on milk, water, or soda in any condition because those products do not typically display those claims or imagery. In condition 5, the FOP percentage juice disclosure was shown on all fruit drinks, 100% juices, and fruit-flavored sodas. All beverages additionally displayed calorie labels in all conditions because that is the current industry standard.

### Outcomes

Primary outcomes were the selection of a high–added sugar beverage in the choice task and selected beverages’ calories and added sugar (in grams) content. We also examined selected beverages’ total sugar (in grams) and the proportion of participants who chose each beverage category: fruit drinks (high, low, and no added sugar), 100% juice, soda, milk, and water.

Secondary outcomes included fruit drink knowledge and perceptions, which were assessed for each drink individually and the mean found for the 2 high–added sugar fruit drinks. Knowledge outcomes included participant estimates of drink juice content (0%-100%; 22 options in 5% intervals) and teaspoons of added sugar/serving (open-text response restricted to 0-100). Estimated juice content values were assigned as the middle value of participant-chosen intervals. We also reported the percentage of participants in each condition who believed the drink was 100% juice.

For perception outcomes, participants rated how much added sugar/serving each drink contained (none, a little, some, or a lot), as well as how likely they were to buy each drink for their child in the next month and how appealing the drink would be to their child based on the packaging (both on a 7-point Likert scale, not at all to extremely). Participants also rated how healthy they thought the drink was for their child (1 = not at all to 7 = extremely) and answered questions about perceived health risks adapted from similar studies.^36,37^ These included whether drinking this drink often would help their child live a healthier life, lead their child to gain excess weight (reverse coded), and increase their child’s risk of dental cavities (reverse coded) (1 = strongly disagree to 7 = strongly agree). These 4 health-related questions were summed to create a health perceptions index (4 = least healthy to 28 = healthiest).

We also assessed perceived message effectiveness (PME),^38^ a scale shown to be sensitive to differences between warnings in online studies and associated with behavior change.^39^ We adapted the scale’s language to assess the entire package instead of a single label on the package. We created a mean PME score as the mean of responses to the following 3 items (1 = strongly disagree to 5 = strongly agree): “This package makes me concerned about the health effects of my child drinking beverages with added sugar”; “This package makes the idea of my child drinking beverages with added sugar seem unpleasant to me”; and “This package discourages me from wanting to let my child drink beverages with added sugar.” We also measured package element recall by assessing whether participants noticed images of fruit, added-sugar warnings, and information about vitamin C, percent juice, or teaspoons of added sugar when selecting a drink for their child.

### Statistical Analysis

Analyses were preregistered with clinicaltrials.gov (NCT04811690) and AsPredicted (trial protocol in Supplement 1; eAppendix 3 in Supplement 2). We tested for differences in participant characteristics across conditions using 2-sided analyses of variance for continuous variables and χ^2^ tests for categorical variables. Linear and logistic regression were used to compare continuous and categorical outcomes, respectively, across conditions. For beverage knowledge and perception outcomes, we prespecified that models would control for the frequency of purchasing each beverage (participants reported how often they had given each brand of drink to their oldest child aged 0-5 years in the last month).

Exploratory analyses examined interactions between condition and potential moderators on selected beverage calories and grams of added sugar using linear regression models with indicators for condition, levels of the potential moderator, and interaction terms between condition and levels of the moderator. Potential moderators tested were educational attainment (≥college vs <college), SNAP status, WIC status, annual household income (≥$75 000 vs <$75 000), frequency of child fruit drink consumption (≥once/wk vs <once/wk), race (Black or other vs White), and Hispanic ethnicity. These race and ethnicity groups were created because we were interested in how outcomes may differ among Black and Hispanic parents compared with White parents. An exploratory logistic regression analysis was also conducted to assess differences in package choice (bottles vs pouches) among participants who chose high–added sugar fruit drinks. All analyses were conducted using Stata/MP statistical software version 17.0 (StataCorp). Statistical significance was set at *P* < .05, and all tests were 2-tailed.

## Results

Among 5005 participants (3587 female participants [71.7%]; mean [SD] age, 31.5 [8.3] years; 2475 White [49.5%], 1154 Black [23.1%], and 1126 Hispanic [22.5%]), there were 714 participants in group 1, 717 participants in group 2, 710 participants in group 3, 717 participants in group 4, 708 participants in group 5, 729 participants in group 6, and 710 participants in group 7. Demographics were balanced across conditions (Table 1). There were 3327 participants (66.5%) without a college education, 2404 participants (48.1%) with an annual household income less than $50 000, 1850 participants (37.0%) who had participated in SNAP in the past year and 1423 participants (28.4%) who had participated in WIC in the last year, and 3368 participants (67.3%) who had young children who consumed fruit drinks once a week or more.

### Beverage Choice

**Figure 3.**
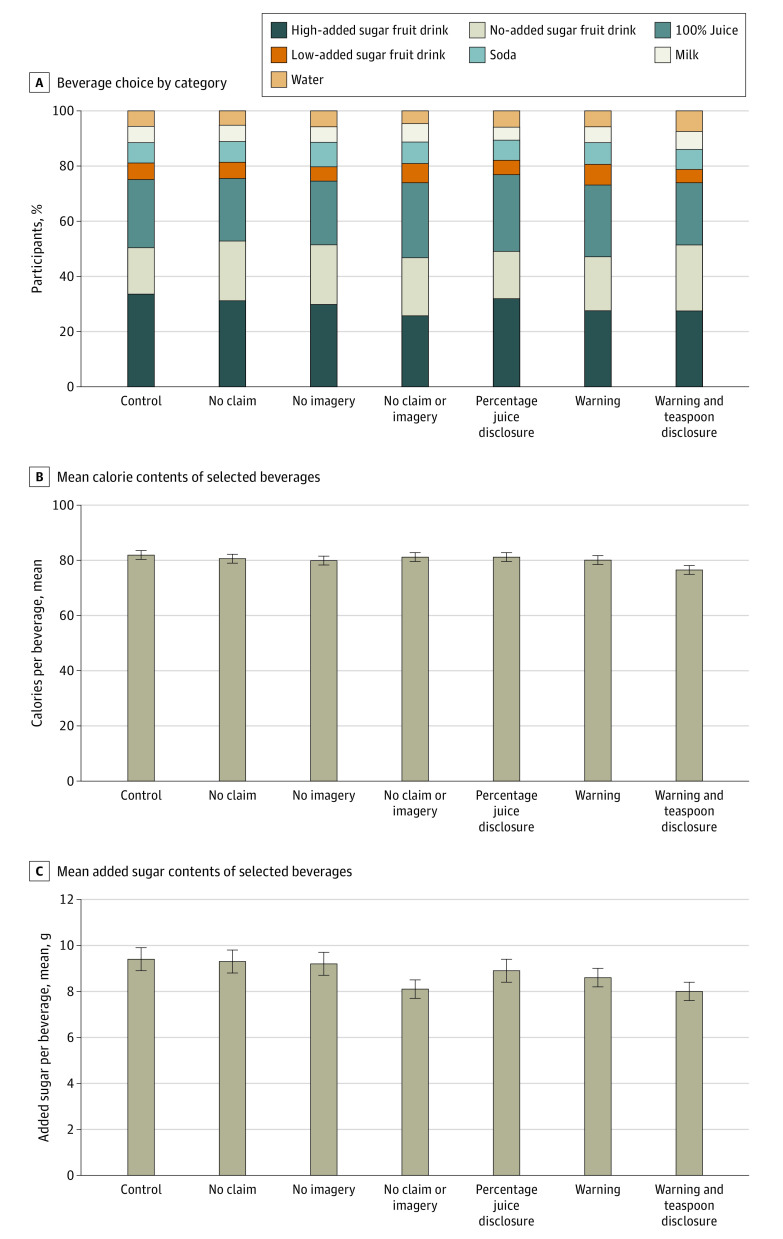
Beverage Choice by Category, Calorie Content, and Total and Added Sugar Content Numerical results are provided in eTable 1 in Supplement 2.

Compared with participants in the control group, among whom a mean (SE) 41.0% (1.8%) chose a high–added sugar beverage, fewer participants chose a high–added sugar beverage for their child in conditions with no 100% vitamin C claim or fruit imagery (absolute difference: −7.6 percentage points; 95% CI, −12.6 to −2.6 percentage points [relative difference: −18.4%; 95% CI, −30.6% to −6.3%]; *P* = .003) and with warnings (high–added sugar warning only: −5.5 percentage points; 95% CI, −10.5 to −0.5 percentage points [−13.4%; 95% CI, −25.6% to −1.2%]; *P* = .03; warning and teaspoons of added sugar disclosure: −6.4 percentage points; 95% CI, −11.4 to −1.4 percentage points [−15.6%; 95% CI, −27.8% to 3.3%]; *P* = .01) (Table 2; eTable 1 in Supplement 2). These results were largely driven by reduced selection of high–added sugar fruit drinks and increased selection of no–added sugar fruit drinks; there were no significant differences in selection of low–added sugar fruit drinks, 100% juice, soda, milk, or water between control and experimental groups (Figure 3A; eTable 1 in Supplement 2). Among participants who chose high–added sugar fruit drinks, selection of bottles vs pouches did not differ between groups (eTable 1 in Supplement 2).

**Table 2.** Effect of Package Modifications on Beverage Choice, Knowledge, Perceptions, and Package Element Recall

Outcome	Control (claim and imagery) (N = 714), mean (SE)	Difference from control (95% CI)					
		No claim (imagery only) (n = 717)	No imagery (claim only) (n = 710)	No claim or imagery (n = 717)	Percentage juice disclosure (n = 708)	Warning (n = 729)	Warning and teaspoon disclosure (n = 710)
Beverage choice							
Chose high–added sugar beverage, % (SE)	41.0 (1.8)	−2.3 (−7.3 to 2.8)	−2.3 (−7.4 to 2.8)	−7.6 (−12.6 to −2.6)	−1.8 (−6.9 to 3.3)	−5.5 (−10.5 to −0.5)	−6.4 (−11.4 to −1.4)
Calories in chosen beverage, kcal	81.9 (1.6)	−1.3 (−5.7 to 3.2)	−2.0 (−6.4 to 2.4)	−0.7 (−5.1 to 3.8)	−0.7 (−5.1 to 3.8)	−1.8 (−6.2 to 2.6)	−5.3 (−9.8 to −0.9)
Added sugar in chosen beverage, g	9.4 (0.5)	−0.1 (−1.3 to 1.2)	−0.2 (−1.4 to 1.1)	−1.2 (−2.5 to 0)	−0.5 (−1.8 to 0.7)	−0.8 (−2.0 to 0.5)	−1.3 (−2.6 to −0.1)
Total sugar in chosen beverage, g	17.7 (0.4)	−0.2 (−1.2 to 0.8)	−0.3 (−1.3 to 0.7)	−0.4 (−1.4 to 0.6)	0 (−1.0 to 1.0)	−0.4 (−1.4 to 0.6)	−1.3 (−2.3 to −0.3)
High–added sugar fruit drink knowledge							
Estimate of % juice, mean (SE)^a^	46.1 (1.0)	−2.7 (−5.5 to 0)	−2.1 (−4.9 to 0.7)	−1.0 (−3.7 to 1.8)	−25.1 (−27.9 to −22.3)	−7.3 (−10.0 to −4.5)	−7.8 (−10.6 to −5.1)
Thought either or both fruit drinks were 100% juice, % (SE)	11.9 (1.2)	−2.5 (−5.7 to 0.7)	1.0 (−2.4 to 4.4)	−0.5 (−3.8 to 2.8)	−9.2 (−11.9 to −6.6)	−2.9 (−6.0 to 0.3)	−2.8 (−6.0 to 0.4)
Added sugar estimate, tsp^b^	5.0 (0.3)	0.3 (−0.6 to 1.1)	0.9 (0 to 1.7)	−0.1 (−1.0 to 0.7)	0.9 (0 to 1.8)	2.6 (1.7 to 3.4)	1.4 (0.6 to 2.3)
High–added sugar fruit drink perceptions							
Added sugar estimate (1-4)^c^	2.8 (0)	0 (−0.1 to 0.1)	0 (−0.1 to 0.1)	0 (−0.1 to 0)	0.1 (0 to 0.2)	0.5 (0.4 to 0.6)	0.5 (0.4 to 0.5)
Likelihood to buy for child (1-7)	4.3 (0)	0 (−0.1 to 0.1)	−0.1 (−0.3 to 0)	−0.1 (−0.3 to 0)	−0.1 (−0.2 to 0)	−0.4 (−0.5 to −0.2)	−0.3 (−0.4 to −0.1)
Likelihood to appeal to child (1-7)	5.0 (0.1)	−0.1 (−0.2 to 0.1)	−0.7 (−0.8 to −0.6)	−0.8 (−0.9 to −0.7)	−0.1 (−0.3 to 0)	−0.3 (−0.5 to −0.2)	−0.2 (−0.3 to −0.1)
Health perception index (4-28)	16.9 (0.2)	−0.3 (−0.7 to 0.2)	−0.1 (−0.6 to 0.3)	−0.1 (−0.5 to 0.4)	−0.8 (−1.2 to −0.4)	−2.3 (−2.7 to −1.8)	−2.1 (−2.5 to −1.7)
Perceived message effectiveness (1-5)	2.7 (0)	0.1 (0 to 0.2)	0.1 (0 to 0.2)	0 (−0.1 to 0.1)	0.2 (0.1 to 0.3)	0.8 (0.7 to 0.9)	0.7 (0.6 to 0.8)
Package element recall, % of sample (SE)							
Images of fruit	93.3 (0.9)	93.3 (0.9)	87.8 (1.2)	87.6 (1.2)	91.7 (1.0)	90.1 (1.1)	91.0 (1.1)
Information about vitamin C	90.6 (1.1)	84.5 (1.4)	87.0 (1.3)	82.2 (1.4)	84.8 (1.4)	85.7 (1.3)	88.7 (1.2)
Information about % juice	36.4 (1.8)	39.8 (1.8)	39.0 (1.8)	37.2 (1.8)	86.3 (1.3)	40.6 (1.8)	43.8 (1.9)
Added sugar warning label	23.3 (1.6)	25.4 (1.6)	23.7 (1.6)	23.4 (1.6)	28.0 (1.7)	71.1 (1.7)	70.9 (1.7)
Information about amount of teaspoons of added sugar	20.5 (1.5)	21.3 (1.5)	20.1 (1.5)	18.8 (1.5)	21.3 (1.5)	23.2 (1.6)	59.9 (1.8)

Abbreviation: SE, standard error.

^a^ The correct mean percentage was 12.5%.

^b^ The correct mean amount was 5 tsp.

^c^ None = 1; a little = 2; some = 3; a lot = 4.

Compared with participants in the control group (who had a mean [SE] of 81.9 (1.6) kcal and 9.4 (0.5) g added sugar in chosen beverages), only participants who saw warnings with teaspoons of added sugar disclosures chose beverages with fewer calories (−5.3 kcal; 95% CI, −9.8 to −0.9 kcal [−6.5%; 95% CI, −11.8% to −1.3%]; *P* = .02) and less added sugar (−1.3 g; 95% CI, −2.6 to −0.1 g [−14.2%; 95% CI, −26.7% to −1.8%]; *P* = .04) and total sugar (−1.3 g; 95% CI, −2.3 to −0.3 g; *P* = .009) for their children (Figure 3B and C). In exploratory analyses, 2 factors modified the effect: WIC participation and Hispanic ethnicity (eFigures 1 and 2 in Supplement 2). Participants who had participated in WIC selected beverages with fewer calories (β = −11.9; *P* for interaction = .02) and added sugar (in grams; β = −3.9; *P* for interaction = .007) than participants who had not participated in WIC when exposed to no claim or imagery compared with participants in the control group. Hispanic participants selected beverages with fewer calories than non-Hispanic participants in the no claim (β = −14.1; *P* for interaction = .009), no imagery (β = −11.4; P for interaction = .03), and warning (β = −13.1; *P* for interaction = .02) conditions compared with participants in the control group.

### High–Added Sugar Fruit Drink Knowledge

Compared with participants in the control group (who estimated a mean [SE] of 46.1% [1.0%] juice), participants in the percentage juice disclosure and both warning conditions had lower (ie, more accurate) percentage juice content estimates for high–added sugar fruit drinks (percentage juice disclosure: −25.1 percentage points; 95% CI, −27.9 to −22.3 percentage points; warning only: −7.3 percentage points; 95% CI, −10.0 to −4.5 percentage points; warning and teaspoons of added sugar disclosure: −7.8 percentage points; 95% CI, −10.6 to −5.1 percentage points; all *P* < .001). Compared with participants in the control group (who estimated a mean [SE] 5.0 [0.3] tsp of added sugar), these groups also estimated more teaspoons of added sugar in high–added sugar fruit drinks (difference from control, percentage juice disclosure: 0.9 tsp; 95% CI, 0 to 1.8 tsp; *P* = .04; warning only: 2.6 tsp; 95% CI, 1.7 to 3.4 tsp; *P* < .001; warning and teaspoons of added sugar disclosure: 1.4 tsp; 95% CI, 0.6 to 2.3 tsp; *P* = .001) (Table 2). Compared with participants in the control group (among whom a mean [SE] 11.9% [1.2%] thought drinks were 100% juice), a smaller proportion of participants in the percentage juice disclosure group thought that high–added sugar fruit drinks were 100% juice (−9.2 percentage points; 95% CI, −11.9 to −6.6 percentage points; *P* < .001). Results for individual drinks are shown in eTable 2 in Supplement 2.

### High–Added Sugar Fruit Drink Perceptions

Compared with participants in the control group, participants in the percentage juice disclosure and both warning conditions perceived high–added sugar fruit drinks to be significantly higher in added sugar and less appealing and healthy (Table 2). Participants in the no claim or imagery and both warning conditions reported a lower likelihood of buying high–added sugar fruit drinks for their children. Participants who viewed high–added sugar fruit drinks without fruit imagery (no imagery or no claim or imagery) perceived those drinks to be significantly less appealing to their children compared with participants in the control group.

### Perceived Message Effectiveness and Package Element Recall

Participants in the percentage juice disclosure and both warning conditions rated high–added sugar fruit drink packages to have significantly higher PME compared with participants in the control group. Most participants reported noticing the package elements they were randomized to see (ranging from 59.9% [SE, 1.8%] for participants in the warning and teaspoon disclosure group seeing information about the amount of teaspoons of added sugar to 93.3% [SE, 0.9%] of participants in the image only group seeing images of fruit) (Table 2).

## Discussion

This randomized clinical trial tested the effects of high–added sugar warnings, percentage juice and teaspoons of added sugar disclosures, and removal of a 100% vitamin C claim or fruit imagery from high–added sugar beverages on parents’ choices for their young children. The warning with teaspoons of added sugar disclosure was the only condition that led parents to select beverages with significantly fewer calories and added sugars. This builds on previous research showing that teaspoon disclosures can reduce sugar in selected beverages.^27,28,29^ We also found that warnings alone and with teaspoons of added sugar disclosures led to 13.4% and 15.6% reductions, respectively, in the proportion of parents selecting high–added sugar beverages for their children. These results are consistent with in-person and online studies of high–added sugar beverage warnings.^22,25^ Adding a percentage juice disclosure, removing a 100% vitamin C claim, or removing fruit imagery had no behavioral effects independently, but the removal of both the claim and imagery resulted in 18.4% fewer parents selecting high–added sugar beverages for their children.

When behavioral effects were observed, parents substituted a no–added sugar fruit drink (not 100% juice, a low–added sugar fruit drink, milk, water, or soda) for high–added sugar fruit drinks. This is a promising finding given that the no–added sugar fruit drink contained less total sugar than 100% juice, no added sugar, and no non-nutritive sweeteners (NNSs). NNSs are not recommended for children^40^ but are often added to fruit drinks,^11^ especially in response to policies requiring high–added sugar warnings.^41,42^ Added-sugar warnings did not increase selection of NNS-containing beverages in this study.

In addition to reducing high–added sugar beverage selection, warnings helped correct participants’ health misperceptions of fruit drinks and resulted in more accurate estimates of percentage juice content, possibly due to lowered health perceptions. Warnings also increased estimates of added sugar content, although this resulted in inaccurate overestimation for 1 of 2 high–added sugar fruit drinks. Consistent with previous research,^22,43^ warnings also decreased participants’ reported likelihood of buying high–added sugar fruit drinks for their children, decreased perceptions of fruit drinks’ healthfulness and appeal, and increased PME.

Although removing both a 100% vitamin C claim and fruit imagery from high–added sugar fruit drinks reduced parents’ selections of high–added sugar drinks, it had no effect on calories. This was because many participants shifted to 100% juice, which contained more calories than some high–added sugar fruit drinks. Claim and imagery removal did not significantly reduce added sugar, which may be due to sample size limitations. Claim and imagery removal had no knowledge or health perception effects combined or independently. While previous findings on whether fruit imagery impacts health perceptions are mixed,^13,44^ our findings differ from previous studies that found that nutrient content claims,^15^ 100% vitamin C claims in particular,^18,44^ increased perceptions that drinks were healthy and selection of high–added sugar beverages. These differences are likely due to product type (branded vs mock or unbranded products) and choices offered (many different beverages vs 1 other beverage). Our findings suggest that regulations restricting both fruit imagery and nutrient content claims may reduce purchases of high–added sugar beverages but that restricting claims or imagery alone may be insufficient.

FOP percentage juice content disclosures did not change behavior in this study, but consistent with prior work,^19^ they resulted in the most accurate estimations of fruit drink juice content and reduced misperceptions that fruit drinks were 100% juice. These disclosures also increased PME and perceived added-sugar content and reduced perceived healthfulness but to a lesser extent than warnings.

Results from our exploratory analyses suggested that warnings and claim or fruit imagery removal may be particularly effective for Hispanic parents, but the driving mechanism remains unknown. While it is possible that individuals with low English language use may rely more on icons and images when making decisions,^45^ Hispanic ethnicity is not necessarily an appropriate proxy for language use. We also found that the removal of both claims and imagery was particularly effective for individuals who participated in WIC, who may associate fruit juice with health because it is provided in WIC food packages.^46^ More research is needed among specific populations to ensure that regulations have equitable impacts.

Strengths of this study include its randomized design in a large, racially and ethnically diverse sample. This study is the first, to our knowledge, to test the effects of FOP percentage juice disclosures and warnings with teaspoons of added sugar disclosures on simulated purchasing behavior. It also is the first, to our knowledge, to compare the effects of adding FOP warnings and disclosures and removing FOP claims and imagery and the first to use real products with branding, claims, and imagery.

### Limitations

This study has several limitations. First, hypothetical purchases were measured, but realistic behavior was incentivized by informing participants that they might receive their selected beverages. However, we did not measure consumption; some parents may not have served an entire resealable fruit drink bottle to their young child at 1 time. Second, social desirability bias may have encouraged participants to select healthier options than they otherwise would have, but this may be similar across randomized groups. Third, we may have been underpowered to detect significant reductions in added sugar between conditions. Fourth, we examined a 1-time exposure to FOP modifications. Future research should test how in-person, repeated exposure to these modifications influences consumer purchasing behavior among different populations. Future research should also explore how such modifications and FOP disclosures of NNS may influence purchases of NNS-containing beverages.

## Conclusions

This randomized clinical trial’s findings suggest that added sugar warnings, especially those that disclose added sugar content in teaspoons, may reduce parents’ purchases of high–added sugar beverages for their young children, improve their understanding of high–added sugar fruit drinks added sugar and percentage juice content, and correct health misperceptions of these drinks. Our findings suggest that prohibiting FOP nutrient claims and fruit imagery on high–added sugar beverages may also reduce parents’ purchases but is unlikely to modify knowledge or perceptions of high–added sugar fruit drinks. Our results also suggest that FOP percentage juice disclosures may improve understanding of juice and added-sugar content but are unlikely to change consumer behavior.

## Notes

Supplement 1.
Trial Protocol and Statistical Analysis Plan

Supplement 2. **eAppendix 1.** Survey Questions **eAppendix 2.** Image Information **eAppendix 3.** Preregistration **eTable 1.** Effect of Front-of-Package Modifications on Beverage Choice **eFigure 1.** Interaction Between Special Supplemental Nutrition Program for Women, Infants, and Children Status and No Claim or Imagery Label Condition on Mean Calories and Added Sugar **eFigure 2.** Interaction Between Hispanic Ethnicity and Label Conditions in Effect on Mean Calories **eTable 2.** Individual Fruit Drink Knowledge and Perceptions

Supplement 3.
Data Sharing Statement
