# Supplementary material for: Effect of Front-of-Package Information, Fruit Imagery, and High–Added Sugar Warning Labels on Parent Beverage Choices for Children: A Randomized Clinical Trial
Source: JAMA Netw Open. 2022 Oct 13;5(10):e2236384. doi: 10.1001/jamanetworkopen.2022.36384 (PMC9561948; doi:10.1001/jamanetworkopen.2022.36384)
Supplement: Supplement 1. — Trial Protocol and Statistical Analysis Plan [file jamanetwopen-e2236384-s001.pdf]

eProtocol: Trial Protocol

Instructions: Complete this template to provide IRB members and designated reviewers with sufficient information to conduct a substantive review of human research. If applicable, submit a Sponsor's Protocol in addition to this document. Detailed instructions for preparing this template can be found in the Investigator's Manual. If the proposed human research is eligible for an Exemption Determination, see Appendix H of the Investigator Manual.

GENERAL INFORMATION	
**Protocol/ESTR Record Number (if assigned): IRB20-2135**	
**Version Number: 4**	**Version Date: 4/27/2021**
**Principal Investigator (PI):** Eric Rimm	
**Principal Investigator's Harvard Affiliation: Faculty**	
**Protocol Title: Testing the Impact of Modifications to Fruit Drink Front-of-Package Label Marketing in an Online Store: A Randomized Controlled Trial**	

1. Specific Aims

Test the independent and combined effects of common front-of-package (FOP) marketing tactics (e.g., 100% Vitamin C claims and fruit images) and added sugar warning labels and nutrition disclosures (e.g., teaspoons of added sugar per serving) on parents' perceptions and beverage choices for their children using a randomized controlled design in a unique online retail setting, and examine differences by race/ethnicity, income, and SNAP/WIC participation.

2. Background and Significance

2.1 Provide the scientific background and rationale for the research.

Fruit drinks are the most widely consumed sugar-sweetened beverages (SSBs) by 0-5-year-olds. Parents purchase these drinks for their children in part due to misperceptions that they are healthful, which may be driven by FOP health claims and imagery. The Food and Drug Administration (FDA) is considering changes to FOP marketing regulations but lacks supporting data on how different marketing tactics influence consumer purchases and perceptions. The proposed research aims to inform federal regulation and fill knowledge gaps on the influence of FOP marketing on behavior and product perceptions, which can then be used to determine effective approaches to correct health misperceptions of fruit drinks.

2.2 Describe the significance of the research, and how it will contribute to generalizable knowledge.

There are large gaps in our knowledge on how FOP marketing tactics on beverages purchased by households with young children affect parents' perceptions and selections, and the effects of changing or countering aspects of FOP marketing to reduce deception or better communicate nutrition information. The majority of prior consumer research on FOP labeling has been conducted in laboratory or online experiments with unbranded beverages; there are limited data on the impact of FOP claims and virtually no data on the impact of FOP imagery on product selection in more realistic retail settings, in which FOP claims, imagery, and branding interact to maximize the influential aspect of the label on purchase behavior. Additionally, there have been no studies to date investigating how legally feasible nutrition warnings and disclosures (e.g., teaspoons of added sugar per serving) could be used with common FOP marketing tactics to correct misperceptions or influence product selections. Preliminary evidence on FOP warning labels suggest they may be effective at reducing SSB purchases, but policy proposals mandating FOP warning labels in the U.S. have been unsuccessful. Disclosures designed to correct misleading advertising may be more legally and politically feasible and as effective, but this has not been studied. Our proposed research contributes to these important knowledge gaps by testing the effects of modifications to existing FOP marketing strategies on parents' perceptions and purchases of SSBs, using a randomized trial in an online store.

Results of this work are timely and can directly inform federal regulation of claims and imagery on food and beverage packaging. The FDA is currently considering changes to claims and other FOP labeling as a part of its Nutrition Innovation Strategy, which aims to reduce preventable death and disease related to poor nutrition, in part by empowering consumers to make better, more informed decisions. The FDA is particularly interested in exploring science-based modifications to claims related to added sugars, especially on products that contain fruit. However, the agency lacks supporting data on how different FOP claims and marketing influence consumers' beverage perceptions and purchase intentions. We have discussed our research intentions with several FDA Senior Advisors for Nutrition Policy, and they have expressed strong support and interest in our work.

3. Research Locations and Collaborating Sites

Research Locations refer to the geographic location that the research will take place, not to the institutions or researchers you may be collaborating with. All Research Locations should be listed in ESTR as a Research Location.

Collaborating Sites refer to institutions or researchers that are also taking part in the research study. All Collaborating Sites should be listed in ESTR as a Participating Site.

3.1. Where will the research activities take place? (check all that apply)

☒ **At Harvard;** *list any non-Harvard Longwood Medical Area (LMA) Schools here: [A list of all Harvard Schools can be found here](#).*

☐ **At another location in Massachusetts;** *specify here:*

☐ **In another state in the U.S.;** *specify here:*

☐ **Internationally;** *specify here:*

3.2. Describe the sites or locations where the research will be conducted or overseen by the Harvard PI. (If conducting the study virtually or remotely, indicate the location of the researcher who is conducting the study.)

Data collection will occur online via Qualtrics. Data will be analyzed by researchers at the Harvard T.H. Chan School of Public Health. Due to COVID restrictions, the researcher analyzing the data will be doing so remotely from Cambridge, MA.

3.3. Describe plans for communication among sites regarding adverse events, interim results, protocol modifications, monitoring of data, etc. ☒ N/A.

3.4. Describe any local (international or state) laws, regulations, and/or customs affecting the research (e.g., age of majority, mandatory reporting requirements, etc). ☒ N/A.

3.5. Identify any approvals or permissions required of collaborating institutions, community leaders, or government officials, including approval from another IRB or local research ethics committee. *Upload copies to the "Study-Related Documents" page in ESTR.* **☒ N/A.**

3.6. Will you collaborate with any researchers not affiliated with Harvard to carry out this study?

☒ **No** ☐ **Yes:** *If yes, list which institutions they are affiliated with. If they are not affiliated with an institution, indicate that here. If yes, also indicate their responsibilities and scope of work in conducting this research.*

3.7. Will your collaborators interact with human subjects, have access to identifiable data/specimens, and/or be responsible for the design, conduct, oversight, or reporting of the research?

☒ **No** ☐ **Yes:** *If yes, indicate if the collaborators will obtain their own IRB review.*

3.8. Will any institution conducting research activities as part of this study, including collaborators, rely on Harvard LMA for IRB review?

☒ **No** ☐ **Yes:** *If yes, list each relying institutions, their site responsible Investigator, and describe what research activities will be conducted there.*

4. Study Team

4.1. Describe the scope of work of the Harvard PI and research team. Indicate who is responsible for the design, conduct, implementation, and/or reporting of the research. Indicate who is responsible for the creation, design, and/or implementation of the study documents/tools.

The Harvard PI, Dr. Eric Rimm, and the project coordinator, Dr. Aviva Musicus, will be the only members of the research team with access to the data. Dr. Rimm is ultimately responsible for the design, conduct, implementation, and reporting of the research. Dr. Musicus is responsible for all data analysis and initial manuscript preparation.

4.2. Describe the Principal Investigator's experience conducting research at the study site(s) and familiarity with the local research context.

Dr. Rimm has previously led research on front-of-package marketing with the research team. Dr. Musicus has extensive research experience conducting randomized controlled trials in an online shopping setting. She has also led numerous studies examining the impact of labeling and marketing on consumer behavior and perceptions using a similar study design to this one.

4.3. Describe how the Principal Investigator will ensure that sufficient time is devoted to conducting and completing the research.

Dr. Rimm has extensive experience leading research studies and working on research teams (see biosketch). Dr. Musicus will serve as project coordinator to assure compliance with IRB protocol, and ensure the study is completed in a timely

manner. She will oversee study administration, dedicating 20 hours/week to the project. Regular check-ins and reporting will ensure the study is conducted and completed on time.

4.4. Describe how all research staff members are trained to ensure that they are adequately informed about the protocol and study-related duties.

Dr. Eric Rimm is the principal investigator on this study, and will oversee study design and analysis. Dr. Aviva Musicus will conduct the data analysis. She is a postdoctoral research fellow in the Department of Social and Behavioral Sciences and has worked with Dr. Rimm on prior research projects. Drs. Rimm and Musicus have reviewed the human research protection program plan and hold current CITI certifications (see attached). They have reviewed the study protocol, and will meet regularly about study-related duties.

4.5. Describe the minimum qualifications for each research role (e.g., RN, social worker, phlebotomist, statistician), their experience in conducting research, and their knowledge of the local research context.

The project coordinator must have experience with data analysis and quantitative research. Dr. Aviva Musicus is a postdoctoral research fellow in the Department of Social and Behavioral Sciences at Harvard Chan and will be coordinating the study and leading data analysis. Dr. Musicus has extensive experience with quantitative research at Harvard, Yale, and with Center for Science in the Public Interest, a non-profit advocacy group in Washington, DC. She has led multiple quantitative research studies.

5. Study Design

5.1. Describe the study design type.

Randomized Controlled Trial

5.2. Does the study involve more than one participant group?

☒ **No** ☐ **Yes:** *If yes, identify each group here and throughout all applicable sections.*

5.3. Indicate the total duration of a participant's involvement.

15-minute online purchasing task and survey

5.4. Indicate the total number of participants to be screened (if applicable) and/or enrolled (i.e., signed consent form). If the proposed research involves secondary data analyses only, indicate the number of data, documents, records, and/or specimens that will be obtained.

5,000 participants will be recruited to take part in our study.

5.5. List inclusion and exclusion criteria, including age ranges of all participants, and describe the screening process. Provide a rationale for any specific exclusion criteria.

We will recruit 5,000 primary caregivers of children 0-5 years old to participate in a randomized controlled online shopping experiment, with oversampling for Hispanic and black participants. We will use Qualtrics, a firm that recruits research participants through their online panels and other online communities, social networks, and websites, to recruit participants with the following eligibility criteria: 1) primary caregiver of a child 0-5 years old; 2) ≥18 years old; and 3) U.S. citizen. For our study, participants will be sent an invitation asking them to "take a survey" to avoid selection bias based on survey topic. Participants will also be required to answer a data integrity check question near the end of the survey (i.e., "what month is it?"). Those who answer incorrectly will be excluded from analyses.

5.6. Describe study procedures.

After providing informed consent, participants will complete a 10-15 minute survey. They will be asked to answer all questions based on their oldest child 0-5 years old, and will provide the age of the child. First, participants will be told to use a mock online retail store to choose a beverage for their child that will be sent to them (or they will receive $5). In reality, at the end of the study, we will debrief participants on the study purpose and inform them that instead of receiving the beverage they selected, all participants will be compensated with $5 through the survey distribution company (twice the value of a typical beverage in the store). This very minor deception enables us to incentivize participants to behave as they normally would when shopping online and to minimize potential social desirability bias introduced by participating in a study. Participants will then be shown images of 12 different beverages (random order) consisting of commonly purchased beverages in various categories, with oversampling for fruit drinks (e.g., 6 fruit drinks, 1 milk, 2 100% juices, 2 regular sodas, and 1 water). The variety will provide a realistic shopping scenario for generalizability. Participants will be required

to click on beverages to see an enlarged package before choosing. After selecting their beverage, participants will advance in the survey to view four randomly ordered fruit drinks one at a time and answer questions about their health knowledge, product beliefs, and purchase intentions. See **Appendix A** for full survey (consent + debriefing language) and **Appendix B** for images.

Label Randomization. Caregivers will be randomized using simple randomization via the Qualtrics platform to see fruit drinks in our online store and survey with one of the following seven label conditions (**Appendix B**). The first condition (*claim & imagery,* control) will represent the status quo and contain a 100% Vitamin C claim and fruit imagery. Conditions 2-4 will involve the removal of package elements for fruit drinks high in added sugar ("high sugar", >=10g/serving, 20% DV): Condition 2 *(imagery only)* will have fruit imagery but no 100% Vitamin C claim on high-sugar fruit drinks; Condition 3 *(claim only)* will have a 100% Vitamin C claim but no fruit imagery on high-sugar fruit drinks; and Condition 4 (*no claim or imagery)* will feature no imagery or claims on high-sugar fruit drinks. Conditions 5-7 will involve the addition of package elements. Condition 5 *(imagery, claim, & text % fruit juice disclosure)* will show a text description of the percent fruit juice in each fruit drink, to see if it changes the effects of the claim and imagery. The sixth *(imagery, claim, & added sugar warning)* and seventh conditions *(imagery, claim, & added sugar warning w/teaspoons of sugar)* will test the effects of added sugar warnings.

Primary Outcomes: The primary outcomes will be total calories and grams of added sugar purchased in the online store task (averaged by condition). We will also examine the percentage of people in each condition that purchased a drink high in added sugars (>20% DV) for their child, and the percentages that chose each category of drink (fruit drinks, 100% juice, etc).

5.7. Does the study involve the use of deception and/or incomplete disclosure?
☐ **No** ☒ **Yes:** *If yes, explain the use of deception/incomplete disclosure and describe why it is necessary to achieve the goals of the study.*

Participants will be told to use the mock online retail space to choose a beverage for their child, and that the beverage will either be sent to them for free or they will receive $5. At the end of the study, we will debrief participants on the study purpose and inform them that all participants receive $5. This very minor deception enables us to incentivize participants to behave as they normally would when shopping online and to minimize potential social desirability bias introduced by participating in a study.

5.8. When all research-related study procedures are complete, are there plans for long-term follow up?
☒ **No** ☐ **Yes:** *If yes, indicate what data will be collected during this period.*

5.9. Does the study involve the collection of specimens (e.g. blood, cells, tissues, fluids, secretions, recombinant or synthetic nucleic acids, biological toxins, bacteria, virus, fungi, etc.)
☒ **No** ☐ **Yes:** *If yes, indicate the COMS Registration Number or plans to obtain COMS approval.*

5.10. Does the study involve the use of existing data, documents, records, and/or specimens for secondary analysis?
☒ **No** ☐ **Yes:** *If yes, indicate how, when, where, and from whom data, documents, records, and/or specimens will be obtained.*

5.11. Are there provisions for medical and/or psychological support resources available to participants (e.g., in the event of incidental findings, research-related stress)?
☒ **No** ☐ **Yes:** *If yes, describe the provisions and their availability.*

5.12. Describe the data and safety monitoring plan for the study. This plan should outline how study progress will be monitored throughout the lifecycle of the research to ensure the safety of subjects, as well as the integrity and confidentiality of data.

Although the survey distribution company will need to collect email addresses to send the $5 incentives to participants, they will remove the email addresses from the dataset before delivering it to us. All data will be delivered to the researchers de-identified with no identifiers other than IP addresses. Confidentiality of data will be ensured by solely keeping data on Dr. Musicus' password-protected work laptop.

5.13. Are there any anticipated circumstances under which participants will be withdrawn from the research without their consent?
☒ **No** ☐ **Yes:** *If yes, describe the circumstances for withdrawal as well any associated procedures to ensure orderly termination, appropriate referrals, and/or follow-up care.*

6. **Recruitment Methods** ☐ **N/A.** *Skip to next section.*
Upload recruitment materials to the "Local-Site Documents" page in ESTR.

6.1. Indicate how, when, where, and by whom participants will be recruited. Provide a list of materials used to recruit participants, e.g., emails, posters, and/or scripts here.

We will use Qualtrics, a leading research firm that recruits research participants through their online panels and other online communities, social networks, and websites, to recruit participants with the following eligibility criteria: 1) primary caregiver of a child 0-5 years old; 2) ≥18 years old; and 3) U.S. citizen. For our study, participants will be sent an invitation asking them to "take a survey" to avoid selection bias based on survey topic.

7. **Consent Process**
Upload consent form(s) and debriefing materials, if applicable, to the "Local-Site Documents" page in ESTR.

7.1. Describe how the research team will invite participants to take part in the research and obtain consent to participate. If the research team will not obtain informed consent, provide justification for requesting a waiver or alteration of consent (and/or parental permission).

Qualtrics will invite participants to take part in the research by sending an invitation asking them to "take a survey". Before conducting the survey, they will be directed to an informed consent page (See **Appendix A**).

7.2. Describe how the research team will document the consent process (e.g., participant/researcher will both sign and date the consent document; participants will thumbprint the consent document; electronic consent will be obtained and associated with the participant's research record). If the research team will not obtain signature and date, provide justification for requesting a waiver or alteration of documentation of consent (and/or parental permission).

Participants will check "yes" to indicate that they have read the informed consent language and wish to proceed to the survey. As the researchers will receive de-identified survey results, we will not obtain a signature and date. The survey will not advance forward if they do not provide informed consent. Participants will be given the option of checking "no" and refusing to take part in the study at any time.

7.3. Will participants be offered a copy of the consent information?
☒ **Yes** ☐ **No:** *If no, explain why not.*

7.4. If consent will be obtained in a language other than English, identify the language(s) that consent information will be provided, who will be responsible for translation, and the provisions for communicating this information to participants. ☒ **N/A**

7.5. If the research involves deception and/or incomplete disclosure, describe the debriefing process. Explain when participants will be debriefed, who will debrief them, and how they will be debriefed. ☐ **N/A**

Participants will receive a debriefing form at the end of the survey explaining the small deception, and they will have the opportunity to check a box if they wish to exclude their data from use in our study (see **Appendix A**).

7.6. If the research involves secondary use of existing data, documents, records, and/or specimens, and the research team will not obtain consent, describe how consent was originally obtained. Additionally, either upload the original consent form to the ESTR record or confirm that the original consent process obtained participants' permission to share or use their data/specimens for future research projects. ☒ **N/A**

8. **HIPAA Privacy Protections** ☒ **N/A.** *Skip to next section.*
HIPAA applies to US-based research involving the collection or use of protected health information (PHI) from a hospital, health center (including the Harvard Dental Center), health plan, or health insurance plan (i.e. a covered entity). The Privacy Rule will not directly regulate researchers who are engaged in research within organizations that are not covered entities even though they may gather, generate, access, and share personal health information (PHI). The Privacy Rule applies only to individually identifiable health information held or maintained by a covered entity. Individually identifiable health information that is held by anyone other than a covered entity, including an independent researcher who is not a covered entity, is not protected by the Privacy Rule and may be used or disclosed without regard to the Privacy Rule.

8.1. Explain how the Privacy Rule applies to this specific research project.

8.2. Describe the covered entity involved in this research that holds or maintains the PHI that will be used by the researchers.

8.3. Describe plans for obtaining authorization to access protected health information or provide the rationale for a waiver of authorization.

9. Research Subject to the European Union (EU) General Data Protection Regulation (GDPR)☒ N/A. *Skip to next section.*
GDPR applies to research involving the collection of "personal data" from research subjects who are located in the EEA. This includes biospecimens. The EU/EEA includes the 28 states of the European Union (Austria, Belgium, Bulgaria, Croatia, Republic of Cyprus, Czech Republic, Denmark, Estonia, Finland, France, Germany, Greece, Hungary, Ireland, Italy, Latvia, Lithuania, Luxembourg, Malta, Netherlands, Poland, Portugal, Romania, Slovakia, Slovenia, Spain, Sweden, & United Kingdom) and four additional countries: Iceland, Liechtenstein, Norway and Switzerland.

9.1. Describe plans to collect and/or obtain "pseudonymized data" (e.g., coded data) and/or identifiable data and/or biospecimens from participants in the EEA.

10. Research Subject to the Family Educational Rights and Privacy Act (FERPA)
☒ N/A. *Skip to next section.*
FERPA applies to research involving the collection of individually identifiable information from student records or personal education information from an education program (defined as: any program principally engaged in the provision of education, including, but not limited to, early childhood education, elementary and secondary education, postsecondary education, special education, job training, career and technical education, and adult education).

10.1. Describe plans to collect and/or obtain individually identifiable information from student records or personal education information from an education program.

11. Vulnerable Populations ☒ N/A. *Skip to next section.*

11.1. Identify all vulnerable populations (e.g., children; pregnant women, human fetuses, neonates; prisoners; elderly; economically disadvantaged; employees or students of the investigator or sponsor; undocumented individuals; refugees; racial and/or ethnic minorities; illiterate or low-literacy; military personnel; terminally ill; cognitively impaired or mentally ill; persons with a stigmatizing disease or condition, e.g. AIDS/HIV, etc.) and describe safeguards to protect their rights and welfare.

12. Risks
Risks may be physical, psychological, social, legal, reputational, and/or financial.

12.1. Describe the reasonably foreseeable risks, discomforts, and/or inconveniences to participants and/or the group/community to which they may belong. Indicate the probability, magnitude, and duration of each risk.

There is a very small risk of breach of data confidentiality, but we expect such a breach would impose no more than minimal risk to study participants. The study involves a very minor deception that enables us to incentivize participants to behave as they normally would when shopping online and to minimize potential social desirability bias introduced by participating in a study. While this may cause brief discomfort to some participants, they were told that they would either receive a drink or $5, so the fact that they receive $5 should not come with too much discomfort.

12.2. Identify whether any of the information collected, if disclosed outside of the research, could reasonably place the participant at risk of criminal or civil liability or be damaging to the participant's financial standing, employability, insurability, or reputation.

None of the information collected would place participants at risk of the above.

12.3. Outline provisions in place to minimize each risk identified above.

No identifiable information besides IP addresses will be delivered to the research team by the survey distribution company. After study completion, IP addresses will be stripped from the dataset so that responses cannot be linked up with this

information. Survey responses will be downloaded from the Qualtrics website and stored on a secure drive. Only the researchers involved in this study will have access to the data.

13. Benefits

13.1. Describe the potential benefits to individual participants, if any, and/or society. If there are no direct benefits, state that here. Note: payment/compensation is not a benefit.

There are no direct benefits to participants. For society, results of this work are timely and can directly inform federal regulation of claims and imagery on food and beverage packaging. The FDA is currently considering changes to claims and other FOP labeling as a part of its Nutrition Innovation Strategy, which aims to reduce preventable death and disease related to poor nutrition, in part by empowering consumers to make better, more informed decisions. The FDA is particularly interested in exploring science-based modifications to claims related to added sugars, especially on products that contain fruit. However, the agency lacks supporting data on how different FOP claims and marketing influence consumers' beverage perceptions and purchase intentions. We have discussed our research intentions with several FDA Senior Advisors for Nutrition Policy, and they have expressed strong support and interest in our work.

14. Participant Privacy

14.1. Describe provisions to protect participants' privacy (their ability to control and limit the extent, timing, and circumstances of sharing information about themselves with others, e.g., the use of a private interview room) and to minimize any sense of intrusiveness that may be caused by study questions or procedures.

Qualtrics will need to collect email addresses from some participants if they wish to receive the extra $5 incentive. These email addresses will solely be used by Qualtrics to send the $5 to participants and then will be destroyed by the survey company. The survey results that Qualtrics will send us will have no identifiable information besides IP addresses. IP addresses are recorded to ensure data quality—duplicate responses from identical IP addresses indicate an individual may have taken the survey twice, and duplicate responses will be removed from the dataset. After study completion, IP addresses will be stripped from the dataset so that responses cannot be linked up with this information. Survey responses will be downloaded from the Qualtrics website and stored on a secure drive. Only the researchers involved in this study will have access to the data.

15. Data Confidentiality

15.1. Indicate the identifiability of the data/specimens:

☐ Data/specimens will not contain any direct or indirect identifiers (anonymous data).

☒ Data/specimens will contain direct or indirect identifiers, but the research team will remove them upon receipt (de-identified data).

☐ Data/specimens will contain indirect identifiers (i.e., number, letter, symbol, or combination thereof) and the research team will maintain a key that links identifiers to individual participants (coded data).

☐ Data/specimens will contain direct identifiers (identifiable data).

☐ None of the above; describe:

15.2. Have any identifiable data/specimens been de-identified for use in this research study?

☐ No ☒ Yes: *If yes, describe how you will prevent any re-identification.*
Qualtrics will need to collect email addresses from some participants if they wish to receive the extra $5 incentive. These email addresses will solely be used by Qualtrics to send the $5 to participants and then will be destroyed by the survey company. The survey results that Qualtrics will send us will have no identifiable information besides IP addresses. IP addresses are recorded to ensure data quality—duplicate responses from identical IP addresses indicate an individual may have taken the survey twice, and duplicate responses will be removed from the dataset. After study completion, IP addresses will be stripped from the dataset so that responses cannot be linked up with this information.

15.3. Identify where data/specimens will be stored (e.g., on campus at Harvard or remotely, in a specimen laboratory) and describe the provisions to maintain confidentiality (e.g., password protection, encryption, locked filing cabinets, etc.). Refer to the Investigator Manual and the Harvard Research Data Security Policy for additional information.

Data will be stored on a Harvard password-protected laptop on the Harvard Chan server in a password-protected folder.

15.4. Indicate whether any data/specimens will be transferred/transmitted and describe the plan to share the data/specimens (e.g., outside of Harvard, to other researchers, to collaborators). Indicate who may request access and how. If data/specimens will be transferred/transmitted/shared, describe how, when, and to whom.

No data will be transmitted or shared with others.

15.5. Indicate whether participants' permission will be obtained to share their data/specimens and/or use their data/specimens in other future research projects.

No data from this study will be used for future research projects.

15.6. Indicate who is responsible for data/specimen management and how the research team and/or other collaborators are permitted access to information.

Dr. Musicus is responsible for data management. The survey is de-identified and no identifiable information besides IP addresses will be given to the researchers from Qualtrics. Survey responses will be downloaded from the Qualtrics website and stored on a secure drive. Only the researchers involved in this study will have access to the data. Dr. Musicus will be the primary holder of the data, and if Dr. Rimm needs access to it, she will share it via a secure file transfer system.

15.7. Indicate how long data/specimens will be stored and describe the plans at the end of the storage period (e.g., are data/specimens destroyed, returned to data/specimen provider, etc.).

De-identified survey results will be stored on a secure server and destroyed 7 years after publication.

16. Data/Statistical Analyses Plan

16.1. Describe plans for analysis (including the statistical method, if applicable).

Linear regression will be used to examine mean total calories and grams of added sugar purchased between our experimental groups. Logistic regression will be used to compare proportions in each condition that chose different beverage types. Because of the randomized design, the only covariates we will control for will be purchasing frequency (for secondary outcomes), and those that may by chance significantly differ among the groups. A Bonferroni-Holm correction will be used to control for multiple comparisons. In secondary analyses we will stratify the main effects by SNAP/WIC status, race/ethnicity, household income, and frequency of child fruit drink consumption.

16.2. Is there a sample size/power calculation?

☐ **No** ☒ **Yes:** *If yes, describe the calculation and the scientific rationale, and, if applicable, by site and key characteristics such as participant demographics.*

To compare the primary outcome of total kcals purchased across the seven groups, we determined that a sample of 4,900 participants (700 per group) would provide at least 80% power to detect an 18-kcal difference between each group, assuming an alpha of 0.05. This estimate is based on effect sizes found in our previous labeling research. It is typical to exclude a small number of participants in online studies (e.g., for failing data integrity check questions), so we will recruit 5,000 participants. Qualtrics has confirmed that this will be feasible given our inclusion criteria.

17. Costs and Compensation ☐ **N/A.** *Skip to next section.*

17.1. Identify any costs that participants may incur during the study, including transportation costs, childcare, or other out-of-pocket expenses.

None

17.2. Identify remuneration that participants may receive during the study. Specify the amount, timing of disbursement, and method (e.g. money, gift cards, in-kind, incentives, raffles, and transportation). Describe how compensation will be calculated and paid if a participant withdraws. If any participant will receive a single payment more than $100, or $600 or more in one calendar year, refer to Harvard University Financial Policy on Human Subject Payments.

Participants will receive a $5 gift card at the end of their participation in the study, dispensed through Qualtrics via participant emails. They will receive compensation regardless of whether they stop the study early.

18. Sharing Study Results ☐ **N/A.** *Skip to next section.*

18.1. Describe the plan to share study results with individual participants, the participant group/community, and/or others.

Results will be published in a scientific journal, and will be shared via webinar with the FDA.

19. Research Related Injuries ☒ **N/A.** *Skip to next section.*

19.1. Describe plans for medical care and compensation for research-related injuries.

20. Reportable Events

20.1. Outline plans for communicating reportable events to the IRB, Sponsor, or others as applicable (e.g., adverse events, unanticipated problems involving risks to participants or others, breach of confidentiality).

Reportable events will be reported within 5 days of the PI finding out in accordance with OHRA policies.

21. Regulatory Compliance

21.1. Describe plans for monitoring regulatory compliance. The monitoring plan should include how you will ensure proper record keeping, retention of required regulatory documents and participant files, and adherence to the IRB-approved protocol and/or IRB policies and procedures. Monitoring plans should describe 1) who is responsible for file maintenance, 2) what will be maintained, 3) how often files will be reviewed and using what method, and 4) where documentation will be retained (for both Regulatory Documents and Participant files).

The study coordinator will be responsible for monitoring regulatory compliance to ensure proper record keeping and retention of required regulatory documents, including the research team CVs and CITI certifications, and IRB submissions (which will also be kept electronically using ESTR). We will use QIP's "CHECKLIST: Investigator Quality Improvement Assessment" to conduct internal checks on a bimonthly basis.

22. Data or Biospecimen Sharing ☒ **N/A.** *Skip to next section.*
If you plan to establish a repository, please submit a separate application using the HLC Repository Protocol Template.

22.1. Describe the plan to send data/specimens to research collaborators outside of Harvard.
☐ **N/A**

22.2. Describe the plan to receive data/specimens from collaborators outside of Harvard.
☐ **N/A**

23. Clinical Trials ☒ **N/A.** *Skip to next section.*
Complete this section for clinical trials, including NIH funded clinical trials or applicable clinical trials (ACT) under the FDA Amendments Act. To determine if a study meets the definition of a clinical trial, follow the guidance in the "Preparing the Research Protocol" section of the Investigator Manual.

23.1. Describe how this study meets the definition of a clinical trial.

23.2. Describe plans for registering this project in a clinical trials registry, e.g., clinicaltrials.gov. If available, provide the registry record number.

23.3. Describe plans for posting the clinical trial consent form on a publicly available federal website per federal requirements in the Common Rule (45§46.116(h)).

24. Device *This section should be completed if the study involves the use of any device on/in/with human subjects, and/or the use any device utilizing human specimens, which meets the FDA definition of a medical device.* ☒ **N/A.** *Skip to next section.*

24.1. Describe the device, including the generic or common name, brand name (if applicable), purpose, function/operation, and whether it is an implant. Indicate who is providing this device for research use.

24.2. Indicate the FDA status of the device as it is being used for the proposed research:
☐ FDA-approved device being used "on-label" (i.e., FDA-approved purpose, population, manner).
☐ FDA-approved device that is being used "off-label" (i.e., for a different purpose, population, or in a different manner than approved).
☐ Not approved by the FDA.

24.3. Indicate the IDE Status of this device:
☐ The use of this device has an IDE.

☐ The use of the device qualifies for an Abbreviated IDE.
☐ The use of the device is exempt from the IDE requirements.

24.4. Has the FDA made a determination as to whether the device is Significant Risk or Non-Significant Risk? ☐ No ☐ Yes: *If yes, indicate the FDA's determination.*

24.5. Describe plans for storage control, and dispending of the product so that (1) only authorized investigators will use the product; (2) the product will only be used in participants who have provided consent, and (3) there will be documented tracking of each product, including unique identifiers and any return/disposal.

25. Drug/Biologic *This section should be completed if the study involves the use of any drug/biologic on/in/with human subjects which meets the FDA definition of a drug/biologic.* ☒ **N/A.** *Skip this section.*

25.1. Describe the drug or biologic, including the generic or common name, brand name (if applicable), dosing, route of administration, number of doses, timing of administration. Indicate who is providing the drug, biologic, supplement for research use.

25.2. Indicate the IND Status of this drug or biologic and who holds the IND: (select one)

☐	There is an IND approval from the FDA for the use of this item.
	The IND is held by:
	The IND number is:
☐	An IND application has been, or will be, submitted to the FDA.
	The IND will be held by:
	The IND number is:
☐	An IND approval is not required.

25.3. Describe how dispensing, delivery and administration will be performed, and by whom. Include information about control (e.g., locked storage), tracking (e.g., lot number, returned pills), documentation, storage, and return/disposal.
