# Supplementary material for: Effect of Front-of-Package Information, Fruit Imagery, and High–Added Sugar Warning Labels on Parent Beverage Choices for Children: A Randomized Clinical Trial
Source: JAMA Netw Open. 2022 Oct 13;5(10):e2236384. doi: 10.1001/jamanetworkopen.2022.36384 (PMC9561948; doi:10.1001/jamanetworkopen.2022.36384)
Supplement: Supplement 2. — eAppendix 1. Survey Questions eAppendix 2. Image Information eAppendix 3. Preregistration eTable 1. Effect of Front-of-Package Modifications on Beverage Choice eFigure 1. Interaction Between Special Supplemental Nutrition Program for Women, Infants, and Children Status and No Claim or Imagery Label Condition on Mean Calories and Added Sugar eFigure 2. Interaction Between Hispanic Ethnicity and Label Conditions in Effect on Mean Calories eTable 2. Individual Fruit Drink Knowledge and Perceptions [file jamanetwopen-e2236384-s002.pdf]

Supplemental Online Content

Musicus AA, Roberto CA, Moran AJ, Sorscher S, Greenthal E, Rimm EB. Effect of front-of-package information, fruit imagery, and high–added sugar warning labels on parent beverage choices for children: a randomized clinical trial. *JAMA Netw Open.* 2022;5(10):e2236384. doi:10.1001/jamanetworkopen.2022.36384

eAppendix 1. Survey Questions
eAppendix 2. Image Information
eAppendix 3. Preregistration
eTable 1. Effect of Front-of-Package Modifications on Beverage Choice
eFigure 1. Interaction Between Special Supplemental Nutrition Program for Women, Infants, and Children Status and No Claim or Imagery Label Condition on Mean Calories and Added Sugar
eFigure 2. Interaction Between Hispanic Ethnicity and Label Conditions in Effect on Mean Calories
eTable 2. Individual Fruit Drink Knowledge and Perceptions

This supplemental material has been provided by the authors to give readers additional information about their work.

© 2022 Musicus AA et al. *JAMA Network Open.*

eAppendix 1. Survey Questions

Construct	Item	Response scale<br>(all force response)
	**A: SCREENING, QUOTAS, CONSENT**	
Screening: Primary Caregiver	Are you a primary caregiver of a child between 0 and 5 years old?<br><br>**[page break]**	1=Yes<br>0=No **[skip to study termination screen]**
Screening: U.S. residence	Do you currently live in the United States of America?<br><br>**[page break]**	1=Yes<br>0=No **[skip to study termination screen]**
Screening: 18 or older	Are you 18 years of age or older?<br><br>**[page break]**	1=Yes<br>0=No **[skip to study termination screen]**
Quota: Race/Ethnicity	Which race(s)/ethnicity(ies) do you consider yourself to be? Check all that apply.<br><br>**[page break]**	1=Asian<br>2=Black or African American<br>3=Latinx/o/a or Hispanic<br>4=Native American<br>5=Native Hawaiian, Pacific Islander, or Alaska Native<br>6=White<br>7=Other<br>**Quotas: No more than White (50%), Black (40%), Hispanic / Latinx (30%)**<br>**[if quota full, skip to study termination screen]**
Quota: Education	What is the highest level of education you have completed? (please check one).<br><br>**[page break]**	1=Less than high school<br>2=High school or received a GED<br>3=Some college (less than 4 years) or Associate's degree (vocational or academic)<br>4=College/university degree (4 years)<br>5=Graduate or professional education (e.g., MA, JD, PhD)<br>**Quotas: High School (no college) (33%), Some College or Associates (33%), Bachelors or Above (33%)**<br>**[if quota full, skip to study termination screen]**

© 2022 Musicus AA et al. *JAMA Network Open.*

Construct	Item	Response scale<br>(all force response)
Consent	You are about to participate in a research study. Participation in this study will involve selecting a drink for your child and answering questions about your attitudes and opinions of different drinks. You will also be asked to provide some information about yourself. The purpose of the study is to understand your attitudes and opinions of different drinks. The survey will take approximately 15 minutes to complete. Your participation in this study is voluntary and you can refuse to take part in the research or stop at any time. Refusal to participate or withdrawal will not involve a penalty or loss of benefits to which you are otherwise entitled.<br><br>The risks associated with your participation in this study are minimal. Although there is the possibility of a breach of confidentiality, the researchers have done what they can to minimize this risk. Researchers will not be able to connect your identity to the questions you answer in the survey. Any information that you provide will not be used or distributed for future research studies, and it will not cost you anything to participate in this research. Society will benefit from this research because it will be used to better understand attitudes and opinions of drinks.<br><br>For completing this survey, you will be compensated with the amount agreed upon when you entered this survey. You will also either receive a multipack of drinks of your choosing or additional compensation worth $5.00.<br><br>If you have any questions, you can email the principal investigator of this study at erimm@hsph.harvard.edu. For questions, concerns, suggestions, or complaints that have not been or cannot be addressed by the researcher, or to report research-related harm, please contact the Office of Regulatory Affairs and Research Compliance at the Harvard T.H. Chan School by calling 1-866-606-0573. If you would like a copy of this consent form, please save this page to your computer.<br><br>Would you like to continue to the survey?<br><br>**[page break]**	1=Yes<br>0=No **[skip to study termination screen]**
	**A2: CHOICE TASK GENERAL INSTRUCTIONS**	

© 2022 Musicus AA et al. *JAMA Network Open.*

Construct	Item	Response scale (all force response)
Prompt	On the next screen you will choose a drink for your child from an online store. At the end of the survey, a computer will randomly select whether we will ship you the drink you selected for free or instead provide you with additional compensation worth $5 for your participation. **If you have more than one child between the ages of 0-5, please choose a drink for your oldest child within that age group.** Note that at times, it might take several seconds for the images to load.<br><br>Please move to the next page to begin.<br><br>**[page break]**	
	**B: BEVERAGE CHOICE TASK**<br>**[In Qualtrics: repeat entire block B x7, one for each label condition]**<br>***See all images used in eSurvey images below***	
Beverage choice task<br>*Primary outcome	On the page below, you will see a variety of drinks. Take a moment to look at your choices, and then click on the drink you would like to purchase for **your oldest child aged 0-5**. **At the end of the survey, a computer will randomly select whether we will ship you the drink you selected for free or instead provide you with additional compensation worth $5 for your participation**. Please make sure to scroll down to see all the drinks before you choose.<br><br>**[page break]**	1=High-added-sugar fruit drink, cranberry-raspberry flavor<br>2=High-added-sugar fruit drink, grape flavor<br>3=Low-added-sugar fruit drink, grape flavor<br>4=High-added-sugar fruit drink, fruit punch flavor<br>5= High-added-sugar fruit drink, pink lemonade flavor<br>6=No-added-sugar fruit drink, fruit punch flavor<br>7=100% Apple Juice<br>8=100% Orange Juice<br>9=Orange Soda<br>10=Cola<br>11=Milk<br>12=Bottled Water<br><br>**[display beverage images from participant's condition, 600 px width, shown in a random arrangement – i.e., choice randomization. All beverages shown were real brand-name products, but brand names are omitted above.]**

© 2022 Musicus AA et al. *JAMA Network Open.*

Construct	Item	Response scale<br>(all force response)
Beverage choice confirmation	<u>**Please confirm your selection**</u> below.<br><br>**[page break]**	1=Confirm **[skip to section C, beverage perceptions task]**<br>2=Go back and select a different drink<br><br>**[display image of beverage selected in previous question, from participant's condition, 600 px width]**
Redo beverage choice [only those who asked to go back and select a different drink]	**[only shown to those who selected "Go back and select a different drink" in previous question]**<br><br>You have indicated that you want a different drink than the one you first chose. Take a moment to look at your choices, and then click on the drink you would like to purchase for <u>your oldest child aged 0-5</u>. As a reminder, <u>**at the end of the survey a computer will randomly select whether we will ship you the drink you selected for free or whether we will instead provide you with additional compensation worth $5 for your participation**</u>. Please make sure to scroll down to see all the drinks before you choose.<br><br>**[page break]**	1=High-added-sugar fruit drink, cranberry-raspberry flavor<br>2=High-added-sugar fruit drink, grape flavor<br>3=Low-added-sugar fruit drink, grape flavor<br>4=High-added-sugar fruit drink, fruit punch flavor<br>5= High-added-sugar fruit drink, pink lemonade flavor<br>6=No-added-sugar fruit drink, fruit punch flavor<br>7=100% Apple Juice<br>8=100% Orange Juice<br>9=Orange Soda<br>10=Cola<br>11=Milk<br>12=Bottled Water<br><br>**[display beverage images from participant's condition, 600 px width, shown in a random arrangement – i.e., choice randomization. All beverages shown were real brand-name products, but brand names are omitted above.]**
	**C: BEVERAGE PERCEPTIONS & KNOWLEDGE TASK**<br>**[In Qualtrics: repeat entire block C x7, one for each label condition; randomize order of display C2/C3/C4/C5]**	
Prompt	You will now view four different images of drinks and answer some questions about them. <u>**When responding to questions about "your child," please think of your oldest child between the ages of 0-5.**</u> The questions will follow each image. Please note that at times, it might take several seconds for the images to load.<br><br>Please move to the next page to begin.<br><br>**[page break]**	

© 2022 Musicus AA et al. *JAMA Network Open.*

Construct	Item	Response scale<br>(all force response)
	**C2: HIGH-ADDED SUGAR FRUIT DRINK PERCEPTIONS & KNOWLEDGE: CRANBERRY-APPLE FLAVOR**	
Prompt & image: high-added-sugar fruit drink	Please view the drink package below and answer the following questions about it.<br><br>**[Display image of high-added-sugar fruit drink, cranberry-apple flavor with label from participant's condition, 600 px width]**	
Likelihood to buy for child: high-added-sugar fruit drink<br><br>*Perceptions*	How likely are you to **buy** this drink for **your child** in the next 4 weeks?	[Horizontal matrix display]<br>1=Not at all 1<br>2=2<br>3=3<br>4=4<br>5=5<br>6=6<br>7=Extremely 7
Appeal to child: high-added-sugar fruit drink<br><br>*Perceptions*	Based on the packaging, how **appealing** would this drink be to **your child**?	[Horizontal matrix display]<br>1=Not at all 1<br>2=2<br>3=3<br>4=4<br>5=5<br>6=6<br>7=Extremely 7
Perceived healthfulness for child, general: high-added-sugar fruit drink<br><br>*Perceptions*	How **healthy** do you think this drink is for **your child**?	[Horizontal matrix display]<br>1=Not at all 1<br>2=2<br>3=3<br>4=4<br>5=5<br>6=6<br>7=Extremely 7
Frequency of child consumption: high-added-sugar fruit drink	How often have you given this brand of drink to **your oldest child aged 0-5** in the last month?	1=Never<br>2=1 time per month<br>3=2-3 times per month<br>4=1-2 times per week<br>5=3-6 times per week<br>6=Every day<br>7=More than once a day
Prompt, perceived healthfulness for child: high-added-sugar fruit drink	Please rate how much you agree or disagree with the following statements about **your oldest child aged 0-5**.<br><br>Drinking this drink often would...	

© 2022 Musicus AA et al. *JAMA Network Open.*

Construct	Item	Response scale<br>(all force response)
Perceived healthfulness for child, weight gain: high-added-sugar fruit drink<br><br>*Perceptions*	...lead my child to **gain excess weight.**	[Horizontal matrix display]<br>1=Strongly Disagree 1<br>2=2<br>3=3<br>4=Neither Agree Nor Disagree 4<br>5=5<br>6=6<br>7=Strongly Agree 7
Perceived healthfulness for child, dental cavities: high-added-sugar fruit drink<br><br>*Perceptions*	...increase my child's risk of **dental cavities.**	[Horizontal matrix display]<br>1=Strongly Disagree 1<br>2=2<br>3=3<br>4=Neither Agree Nor Disagree 4<br>5=5<br>6=6<br>7=Strongly Agree 7
Perceived healthfulness for child, healthier life: high-added-sugar fruit drink<br><br>*Perceptions*	...help my child live a **healthier life.**<br><br>**[page break]**	[Horizontal matrix display]<br>1=Strongly Disagree 1<br>2=2<br>3=3<br>4=Neither Agree Nor Disagree 4<br>5=5<br>6=6<br>7=Strongly Agree 7
Prompt & image: high-added-sugar fruit drink	Please view the drink package below and answer the following questions about it.<br><br>**[Display image of high-added-sugar fruit drink, cranberry-apple flavor with label from participant’s condition, 600 px width]**	
Perceived Message Effectiveness (PME), health concern: high-added-sugar fruit drink<br><br>*Perceptions*	This package makes me **<u>concerned about the health effects</u>** of my child drinking beverages with added sugar.	1=Strongly Disagree 1<br>2=Somewhat Disagree 2<br>3=Neither Agree Nor Disagree 3<br>4=Somewhat Agree 4<br>5=Strongly Agree 5
Perceived Message Effectiveness (PME), unpleasant: high-added-sugar fruit drink<br><br>*Perceptions*	This package makes the idea of my child drinking beverages with added sugar **<u>seem unpleasant</u>** to me.	1=Strongly Disagree 1<br>2=Somewhat Disagree 2<br>3=Neither Agree Nor Disagree 3<br>4=Somewhat Agree 4<br>5=Strongly Agree 5

© 2022 Musicus AA et al. *JAMA Network Open.*

Construct	Item	Response scale (all force response)
Perceived Message Effectiveness (PME), discourage: high-added-sugar fruit drink<br><br>*Perceptions*	This package **discourages me from wanting to let my child drink** beverages with added sugar.<br><br>**[page break]**	1=Strongly Disagree 1<br>2=Somewhat Disagree 2<br>3=Neither Agree Nor Disagree 3<br>4=Somewhat Agree 4<br>5=Strongly Agree 5
Prompt & image: high-added-sugar fruit drink	Please view the drink package below and answer the following questions about it.<br><br>**[Display image of high-added-sugar fruit drink, cranberry-apple flavor with label from participant's condition, 600 px width]**	
Percent juice estimate: high-added-sugar fruit drink<br><br>*Knowledge*	How much **juice** do you think this product has?	[Drop-down menu display]<br>0=0% (None)<br>1=1 – 4%<br>2=5 – 9%<br>3=10 – 14%<br>4=15 – 19%<br>5=20 – 24%<br>6=25 – 29%<br>7=30 – 34%<br>8=35 – 39%<br>9=40 – 44%<br>10=45 – 49%<br>11=50 – 54%<br>12=55 – 59%<br>13=60 – 64%<br>14=65 – 69%<br>15=70 – 74%<br>16=75 – 79%<br>17=80 – 84%<br>18=85 – 89%<br>19=90 – 94%<br>20=95 – 99%<br>21=100%
Added sugar estimate, categorical: high-added-sugar fruit drink<br><br>*Perceptions*	How much **added sugar** do you think is in one serving of this drink (10 oz bottle)?	1=None<br>2=A little<br>3=Some<br>4=A lot
Added sugar estimate, tsp: high-added-sugar fruit drink<br><br>*Knowledge*	How many **teaspoons of added sugar** do you think is in one serving of this drink (10 oz bottle)? If you're not sure, make your best guess.<br><br>**[page break]**	[free response, #, restricted to 0–100]
	**C3: HIGH-ADDED SUGAR FRUIT DRINK PERCEPTIONS & KNOWLEDGE: STRAWBERRY KIWI FLAVOR**	

© 2022 Musicus AA et al. *JAMA Network Open.*

Construct	Item	Response scale<br>(all force response)
Prompt & image: high-added-sugar fruit drink	Please view the drink package below and answer the following questions about it.<br><br>**[Display image of high-added-sugar fruit drink, strawberry-kiwi flavor with label from participant's condition, 600 px width]**	
Likelihood to buy for child: high-added-sugar fruit drink<br><br>*Perceptions*	How likely are you to **buy** this drink for **your child** in the next 4 weeks?	[Horizontal matrix display]<br>1=Not at all 1<br>2=2<br>3=3<br>4=4<br>5=5<br>6=6<br>7=Extremely 7
Appeal to child: high-added-sugar fruit drink<br><br>*Perceptions*	Based on the packaging, how **appealing** would this drink be to **your child**?	[Horizontal matrix display]<br>1=Not at all 1<br>2=2<br>3=3<br>4=4<br>5=5<br>6=6<br>7=Extremely 7
Perceived healthfulness for child, general: high-added-sugar fruit drink<br><br>*Perceptions*	How **healthy** do you think this drink is for **your child**?	[Horizontal matrix display]<br>1=Not at all 1<br>2=2<br>3=3<br>4=4<br>5=5<br>6=6<br>7=Extremely 7
Frequency of child consumption: high-added-sugar fruit drink	How often have you given this brand of drink to **your oldest child aged 0-5** in the last month?	1=Never<br>2=1 time per month<br>3=2-3 times per month<br>4=1-2 times per week<br>5=3-6 times per week<br>6=Every day<br>7=More than once a day
Prompt, perceived healthfulness for child: high-added-sugar fruit drink	Please rate how much you agree or disagree with the following statements about **your oldest child aged 0-5**.<br><br>Drinking this drink often would...	

© 2022 Musicus AA et al. *JAMA Network Open.*

Construct	Item	Response scale<br>(all force response)
Perceived healthfulness for child, weight gain: high-added-sugar fruit drink<br><br>*Perceptions*	...lead my child to **gain excess weight.**	[Horizontal matrix display]<br>1=Strongly Disagree 1<br>2=2<br>3=3<br>4=Neither Agree Nor Disagree 4<br>5=5<br>6=6<br>7=Strongly Agree 7
Perceived healthfulness for child, dental cavities: high-added-sugar fruit drink<br><br>*Perceptions*	...increase my child's risk of **dental cavities.**	[Horizontal matrix display]<br>1=Strongly Disagree 1<br>2=2<br>3=3<br>4=Neither Agree Nor Disagree 4<br>5=5<br>6=6<br>7=Strongly Agree 7
Perceived healthfulness for child, healthier life: high-added-sugar fruit drink<br><br>*Perceptions*	...help my child live a **healthier life.**<br><br>**[page break]**	[Horizontal matrix display]<br>1=Strongly Disagree 1<br>2=2<br>3=3<br>4=Neither Agree Nor Disagree 4<br>5=5<br>6=6<br>7=Strongly Agree 7
Prompt & image: high-added-sugar fruit drink	Please view the drink package below and answer the following questions about it.<br><br>**[Display image of high-added-sugar fruit drink, strawberry-kiwi with label from participant's condition, 600 px width]**	
Perceived Message Effectiveness (PME), health concern: high-added-sugar fruit drink<br><br>*Perceptions*	This package makes me **<u>concerned about the health effects</u>** of my child drinking beverages with added sugar.	1=Strongly Disagree 1<br>2=Somewhat Disagree 2<br>3=Neither Agree Nor Disagree 3<br>4=Somewhat Agree 4<br>5=Strongly Agree 5
Perceived Message Effectiveness (PME), unpleasant: high-added-sugar fruit drink<br><br>*Perceptions*	This package makes the idea of my child drinking beverages with added sugar **<u>seem unpleasant</u>** to me.	1=Strongly Disagree 1<br>2=Somewhat Disagree 2<br>3=Neither Agree Nor Disagree 3<br>4=Somewhat Agree 4<br>5=Strongly Agree 5

© 2022 Musicus AA et al. *JAMA Network Open.*

Construct	Item	Response scale<br>(all force response)
Perceived Message Effectiveness (PME), discourage: high-added-sugar fruit drink<br><br>*Perceptions*	This package **discourages me from wanting to let my child drink** beverages with added sugar.<br><br>**[page break]**	1=Strongly Disagree 1<br>2=Somewhat Disagree 2<br>3=Neither Agree Nor Disagree 3<br>4=Somewhat Agree 4<br>5=Strongly Agree 5
Prompt & image: high-added-sugar fruit drink	Please view the drink package below and answer the following questions about it.<br><br>**[Display image of high-added-sugar fruit drink, strawberry-kiwi with label from participant's condition, 600 px width]**	
Percent juice estimate: high-added-sugar fruit drink<br><br>*Knowledge*	How much **juice** do you think this product has?	[Drop-down menu display]<br>0=0% (None)<br>1=1 – 4%<br>2=5 – 9%<br>3=10 – 14%<br>4=15 – 19%<br>5=20 – 24%<br>6=25 – 29%<br>7=30 – 34%<br>8=35 – 39%<br>9=40 – 44%<br>10=45 – 49%<br>11=50 – 54%<br>12=55 – 59%<br>13=60 – 64%<br>14=65 – 69%<br>15=70 – 74%<br>16=75 – 79%<br>17=80 – 84%<br>18=85 – 89%<br>19=90 – 94%<br>20=95 – 99%<br>21=100%
Added sugar estimate, categorical: high-added-sugar fruit drink<br><br>*Perceptions*	How much **added sugar** do you think is in one serving of this drink (6 oz pouch)?	1=None<br>2=A little<br>3=Some<br>4=A lot
Added sugar estimate, tsp: high-added-sugar fruit drink<br><br>*Knowledge*	How many **teaspoons of added sugar** do you think is in one serving of this drink (6 oz pouch)? If you're not sure, make your best guess.<br><br>**[page break]**	[free response, #, restricted to 0–100]
	**C4: LOW-ADDED SUGAR FRUIT DRINK PERCEPTIONS & KNOWLEDGE: ORANGE FLAVOR**	

© 2022 Musicus AA et al. *JAMA Network Open.*

Construct	Item	Response scale<br>(all force response)
Prompt & image: low-added-sugar fruit drink	Please view the drink package below and answer the following questions about it.<br><br>**[Display image of low-added-sugar fruit drink, orange flavor with label from participant's condition, 600 px width]**	
Likelihood to buy for child: low-added-sugar fruit drink<br><br>*Perceptions*	How likely are you to **buy** this drink for **your child** in the next 4 weeks?	[Horizontal matrix display]<br>1=Not at all 1<br>2=2<br>3=3<br>4=4<br>5=5<br>6=6<br>7=Extremely 7
Appeal to child: low-added-sugar fruit drink<br><br>*Perceptions*	Based on the packaging, how **appealing** would this drink be to **your child**?	[Horizontal matrix display]<br>1=Not at all 1<br>2=2<br>3=3<br>4=4<br>5=5<br>6=6<br>7=Extremely 7
Perceived healthfulness for child, general: low-added-sugar fruit drink<br><br>*Perceptions*	How **healthy** do you think this drink is for **your child**?	[Horizontal matrix display]<br>1=Not at all 1<br>2=2<br>3=3<br>4=4<br>5=5<br>6=6<br>7=Extremely 7
Frequency of child consumption: low-added-sugar fruit drink	How often have you given this brand of drink to **your oldest child aged 0-5** in the last month?	1=Never<br>2=1 time per month<br>3=2-3 times per month<br>4=1-2 times per week<br>5=3-6 times per week<br>6=Every day<br>7=More than once a day
Prompt, perceived healthfulness for child: low-added-sugar fruit drink	Please rate how much you agree or disagree with the following statements about **your oldest child aged 0-5**.<br><br>Drinking this drink often would...	

© 2022 Musicus AA et al. *JAMA Network Open.*

Construct	Item	Response scale<br>(all force response)
Perceived healthfulness for child, weight gain: low-added-sugar fruit drink<br><br>*Perceptions*	...lead my child to **gain excess weight.**	[Horizontal matrix display]<br>1=Strongly Disagree 1<br>2=2<br>3=3<br>4=Neither Agree Nor Disagree 4<br>5=5<br>6=6<br>7=Strongly Agree 7
Perceived healthfulness for child, dental cavities: low-added-sugar fruit drink<br><br>*Perceptions*	...increase my child's risk of **dental cavities.**	[Horizontal matrix display]<br>1=Strongly Disagree 1<br>2=2<br>3=3<br>4=Neither Agree Nor Disagree 4<br>5=5<br>6=6<br>7=Strongly Agree 7
Perceived healthfulness for child, healthier life: low-added-sugar fruit drink<br><br>*Perceptions*	...help my child live a **healthier life.**<br><br>**[page break]**	[Horizontal matrix display]<br>1=Strongly Disagree 1<br>2=2<br>3=3<br>4=Neither Agree Nor Disagree 4<br>5=5<br>6=6<br>7=Strongly Agree 7
Prompt & image: low-added-sugar fruit drink	Please view the drink package below and answer the following questions about it.<br><br>**[Display image of low-added-sugar fruit drink, orange flavor with label from participant's condition, 600 px width]**	
Perceived Message Effectiveness (PME), health concern: low-added-sugar fruit drink<br><br>*Perceptions*	This package makes me <u>**concerned about the health effects**</u> of my child drinking beverages with added sugar.	1=Strongly Disagree 1<br>2=Somewhat Disagree 2<br>3=Neither Agree Nor Disagree 3<br>4=Somewhat Agree 4<br>5=Strongly Agree 5
Perceived Message Effectiveness (PME), unpleasant: low-added-sugar fruit drink<br><br>*Perceptions*	This package makes the idea of my child drinking beverages with added sugar <u>**seem unpleasant**</u> to me.	1=Strongly Disagree 1<br>2=Somewhat Disagree 2<br>3=Neither Agree Nor Disagree 3<br>4=Somewhat Agree 4<br>5=Strongly Agree 5

© 2022 Musicus AA et al. *JAMA Network Open.*

Construct	Item	Response scale<br>(all force response)
Perceived Message Effectiveness (PME), discourage: low-added-sugar fruit drink<br><br>*Perceptions*	This package **discourages me from wanting to let my child drink** beverages with added sugar.<br><br>**[page break]**	1=Strongly Disagree 1<br>2=Somewhat Disagree 2<br>3=Neither Agree Nor Disagree 3<br>4=Somewhat Agree 4<br>5=Strongly Agree 5
Prompt & image: low-added-sugar fruit drink	Please view the drink package below and answer the following questions about it.<br><br>**[Display image of low-added-sugar fruit drink, orange flavor with label from participant's condition, 600 px width]**	
Percent juice estimate: low-added-sugar fruit drink<br><br>*Knowledge*	How much **juice** do you think this product has?	[Drop-down menu display]<br>0=0% (None)<br>1=1 – 4%<br>2=5 – 9%<br>3=10 – 14%<br>4=15 – 19%<br>5=20 – 24%<br>6=25 – 29%<br>7=30 – 34%<br>8=35 – 39%<br>9=40 – 44%<br>10=45 – 49%<br>11=50 – 54%<br>12=55 – 59%<br>13=60 – 64%<br>14=65 – 69%<br>15=70 – 74%<br>16=75 – 79%<br>17=80 – 84%<br>18=85 – 89%<br>19=90 – 94%<br>20=95 – 99%<br>21=100%
Added sugar estimate, categorical: low-added-sugar fruit drink<br><br>*Perceptions*	How much **added sugar** do you think is in one serving of this drink (6 oz box)?	1=None<br>2=A little<br>3=Some<br>4=A lot
Added sugar estimate, tsp: low-added-sugar fruit drink<br><br>*Knowledge*	How many **teaspoons of added sugar** do you think is in one serving of this drink (6 oz box)? If you're not sure, make your best guess.<br><br>**[page break]**	[free response, #, restricted to 0–100]
	**C5: NO-ADDED SUGAR JUICE DRINK PERCEPTIONS & KNOWLEDGE: STRAWBERRY PEACH FLAVOR**	

© 2022 Musicus AA et al. *JAMA Network Open.*

Construct	Item	Response scale (all force response)
Prompt & image: no-added-sugar juice drink	Please view the drink package below and answer the following questions about it.<br><br>**[Display image of no-added-sugar fruit drink, strawberry peach flavor with label from participant's condition, 600 px width]**	
Likelihood to buy for child: no-added-sugar juice drink<br><br>*Perceptions*	How likely are you to **buy** this drink for **your child** in the next 4 weeks?	[Horizontal matrix display]<br>1=Not at all 1<br>2=2<br>3=3<br>4=4<br>5=5<br>6=6<br>7=Extremely 7
Appeal to child: no-added-sugar juice drink<br><br>*Perceptions*	Based on the packaging, how **appealing** would this drink be to **your child**?	[Horizontal matrix display]<br>1=Not at all 1<br>2=2<br>3=3<br>4=4<br>5=5<br>6=6<br>7=Extremely 7
Perceived healthfulness for child, general: no-added-sugar juice drink<br><br>*Perceptions*	How **healthy** do you think this drink is for **your child**?	[Horizontal matrix display]<br>1=Not at all 1<br>2=2<br>3=3<br>4=4<br>5=5<br>6=6<br>7=Extremely 7
Frequency of child consumption: no-added-sugar juice drink	How often have you given this brand of drink to **your oldest child aged 0-5** in the last month?	1=Never<br>2=1 time per month<br>3=2-3 times per month<br>4=1-2 times per week<br>5=3-6 times per week<br>6=Every day<br>7=More than once a day
Prompt, perceived healthfulness for child: no-added-sugar juice drink	Please rate how much you agree or disagree with the following statements about **your oldest child aged 0-5**.<br><br>Drinking this drink often would...	

© 2022 Musicus AA et al. *JAMA Network Open.*

Construct	Item	Response scale<br>(all force response)
Perceived healthfulness for child, weight gain: no-added-sugar juice drink<br><br>*Perceptions*	...lead my child to **gain excess weight.**	[Horizontal matrix display]<br>1=Strongly Disagree 1<br>2=2<br>3=3<br>4=Neither Agree Nor Disagree 4<br>5=5<br>6=6<br>7=Strongly Agree 7
Perceived healthfulness for child, dental cavities: no-added-sugar juice drink<br><br>*Perceptions*	...increase my child's risk of **dental cavities.**	[Horizontal matrix display]<br>1=Strongly Disagree 1<br>2=2<br>3=3<br>4=Neither Agree Nor Disagree 4<br>5=5<br>6=6<br>7=Strongly Agree 7
Perceived healthfulness for child, healthier life: no-added-sugar juice drink<br><br>*Perceptions*	...help my child live a **healthier life.**<br><br>**[page break]**	[Horizontal matrix display]<br>1=Strongly Disagree 1<br>2=2<br>3=3<br>4=Neither Agree Nor Disagree 4<br>5=5<br>6=6<br>7=Strongly Agree 7
Prompt & image: no-added-sugar juice drink	Please view the drink package below and answer the following questions about it.<br><br>**[Display image of no-added-sugar fruit drink, strawberry peach flavor with label from participant's condition, 600 px width]**	
Perceived Message Effectiveness (PME), health concern: no-added-sugar juice drink<br><br>*Perceptions*	This package makes me **concerned about the health effects** of my child drinking beverages with added sugar.	1=Strongly Disagree 1<br>2=Somewhat Disagree 2<br>3=Neither Agree Nor Disagree 3<br>4=Somewhat Agree 4<br>5=Strongly Agree 5
Perceived Message Effectiveness (PME), unpleasant: no-added-sugar juice drink<br><br>*Perceptions*	This package makes the idea of my child drinking beverages with added sugar **seem unpleasant** to me.	1=Strongly Disagree 1<br>2=Somewhat Disagree 2<br>3=Neither Agree Nor Disagree 3<br>4=Somewhat Agree 4<br>5=Strongly Agree 5

© 2022 Musicus AA et al. *JAMA Network Open.*

Construct	Item	Response scale<br>(all force response)
Perceived Message Effectiveness (PME), discourage: no-added-sugar juice drink<br><br>*Perceptions*	This package **discourages me from wanting to let my child drink** beverages with added sugar.<br><br>**[page break]**	1=Strongly Disagree 1<br>2=Somewhat Disagree 2<br>3=Neither Agree Nor Disagree 3<br>4=Somewhat Agree 4<br>5=Strongly Agree 5
Prompt & image: no-added-sugar juice drink	Please view the drink package below and answer the following questions about it.<br><br>**[Display image of no-added-sugar fruit drink, strawberry peach flavor with label from participant's condition, 600 px width]**	
Percent juice estimate: no-added-sugar juice drink<br>*Knowledge*	How much **juice** do you think this product has?	[Drop-down menu display]<br>0=0% (None)<br>1=1 – 4%<br>2=5 – 9%<br>3=10 – 14%<br>4=15 – 19%<br>5=20 – 24%<br>6=25 – 29%<br>7=30 – 34%<br>8=35 – 39%<br>9=40 – 44%<br>10=45 – 49%<br>11=50 – 54%<br>12=55 – 59%<br>13=60 – 64%<br>14=65 – 69%<br>15=70 – 74%<br>16=75 – 79%<br>17=80 – 84%<br>18=85 – 89%<br>19=90 – 94%<br>20=95 – 99%<br>21=100%
Added sugar estimate, categorical: no-added-sugar juice drink<br><br>*Perceptions*	How much **added sugar** do you think is in one serving of this drink (6 oz box)?	1=None<br>2=A little<br>3=Some<br>4=A lot
Added sugar estimate, tsp: no-added-sugar juice drink<br><br>*Knowledge*	How many **teaspoons of added sugar** do you think is in one serving of this drink (6 oz box)? If you're not sure, make your best guess.<br><br>**[page break]**	[free response, #, restricted to 0–100]
	**D: PACKAGE ELEMENT RECALL**	

© 2022 Musicus AA et al. *JAMA Network Open.*

Construct	Item	Response scale<br>(all force response)
Prompt, matrix	Think back to the beginning of this survey. When you were selecting a drink for your child, did you notice any of the following package elements on any of the drinks?	
Recall, imagery	Images of fruit	[Horizontal matrix display]<br>0=No<br>1=Yes **[display follow-up influence question]**<br>2=I don't remember
Recall, vitamin C	Information about vitamin C	[Horizontal matrix display]<br>0=No<br>1=Yes **[display follow-up influence question]**<br>2=I don't remember
Recall, percent juice	Information about % juice in the drink	[Horizontal matrix display]<br>0=No<br>1=Yes **[display follow-up influence question]**<br>2=I don't remember
Recall, added sugars warning	Added sugars warning label	[Horizontal matrix display]<br>0=No<br>1=Yes **[display follow-up influence question]**<br>2=I don't remember
Recall, teaspoons added sugar	Amount of teaspoons of added sugar in the drink<br><br>**[page break]**	[Horizontal matrix display]<br>0=No<br>1=Yes **[display follow-up influence question]**<br>2=I don't remember
Influence, imagery	**[only shown to those who selected "yes" to Recall, imagery question]**<br><br>How much did **images of fruit** influence which drink you chose, if at all?<br><br>**[page break]**	1=Made me **more likely** to choose a drink with that package element<br>2= Made me **less likely** to choose a drink with that package element<br>3=Had **no effect** on what I chose
Influence, vitamin C	**[only shown to those who selected "yes" to Recall, vitamin C question]**<br><br>How much did **information about vitamin C** influence which drink you chose, if at all?<br><br>**[page break]**	1=Made me **more likely** to choose a drink with that package element<br>2= Made me **less likely** to choose a drink with that package element<br>3=Had **no effect** on what I chose

© 2022 Musicus AA et al. *JAMA Network Open.*

Construct	Item	Response scale<br>(all force response)
Influence, percent juice	**[only shown to those who selected "yes" to Recall, percent juice question]**<br><br>How much did **information about % juice in the drink** influence which drink you chose, if at all?<br><br>**[page break]**	1=Made me **more likely** to choose a drink with that package element<br>2= Made me **less likely** to choose a drink with that package element<br>3=Had **no effect** on what I chose
Influence, added sugars warning	**[only shown to those who selected "yes" to Recall, added sugars warning question]**<br><br>How much did an **added sugars warning label** influence which drink you chose, if at all?<br><br>**[page break]**	1=Made me **more likely** to choose a drink with that package element<br>2= Made me **less likely** to choose a drink with that package element<br>3=Had **no effect** on what I chose
Influence, teaspoons added sugar	**[only shown to those who selected "yes" to Recall, teaspoons added sugar question]**<br><br>How much did seeing **the amount of teaspoons of added sugar in the drink** influence which drink you chose, if at all?<br><br>**[page break]**	1=Made me **more likely** to choose a drink with that package element<br>2= Made me **less likely** to choose a drink with that package element<br>3=Had **no effect** on what I chose
	**E: DEMOGRAPHICS**	
Prompt	You're almost done! We just need to ask you a few questions about yourself.	
Age	What is your age?	[free response, #, restricted to 18–100]
Gender	What is your gender? Select the option that best describes your gender.	1=Woman<br>2=Man<br>3=Neither woman nor man<br>4=Prefer to self-describe:_____<br>5=Prefer not to say
Height, feet	How tall are you (in feet and inches)?<br><br>**[broken up into two constructs, one for feet, one for inches]**	[Drop-down menu display]<br>3=3 feet<br>4=4 feet<br>5=5 feet<br>6=6 feet<br>7=7 feet

© 2022 Musicus AA et al. *JAMA Network Open.*

Construct	Item	Response scale<br>(all force response)
Height, inches	**[Show on same page as above – separate drop-down menu for inches]**	[Drop-down menu display]<br>0=0 inches<br>1=1 inches<br>2=2 inches<br>3=3 inches<br>4=4 inches<br>5=5 inches<br>6=6 inches<br>7=7 inches<br>8=8 inches<br>9=9 inches<br>10=10 inches<br>11=11 inches
Weight	What is your weight? (in pounds)	[free response, #, restricted to 20–700]
Number of children	How many children do you have?	[free response, #, restricted to 0–20]
Child age	How old is your oldest child in the age range of 0 to 5?	0=Younger than 1 year old<br>1=1 year old<br>2=2 years old<br>3=3 years old<br>4=4 years old<br>5=5 years old
Frequency of child fruit drink consumption	How often does your oldest child aged 0-5 drink fruit drinks? These include any fruit-flavored drinks (liquid or powder) that are not 100% juice, such as Capri Sun, Kool-Aid, or juice cocktails.	1=Never<br>2=1 time per month<br>3=2-3 times per month<br>4=1-2 times per week<br>5=3-6 times per week<br>6=Every day<br>7=More than once a day
Diagnosed health conditions	Have you ever been diagnosed with the following by a health professional? Check all that apply.	1=Pre-diabetes<br>2=Diabetes<br>3=Obesity<br>4=None of the above

© 2022 Musicus AA et al. *JAMA Network Open.*

Construct	Item	Response scale<br>(all force response)
Attention check	What month is it?	1=January<br>2=February<br>3=March<br>4=April<br>5=May<br>6=June<br>7=July<br>8=August<br>9=September<br>10=October<br>11=November<br>12=December
SNAP participation last 12 months	In the last 12 months, did you receive benefits from SNAP (the Supplemental Nutrition Assistance Program), formerly known as the Food Stamp Program?	1=Yes<br>0=No
WIC participation last 12 months	In the last 12 months, did you receive benefits from the WIC program (the Special Supplemental Nutrition Program for Women, Infants, and Children)?	1=Yes<br>0=No
Annual household income	What is the combined annual income of the adults (i.e., you and your partner, if applicable) in your household before taxes?	1=Less than $25,000<br>2=$25,000 – $49,999<br>3=$50,000 – $74,999<br>4=$75,000 – $99,999<br>5=$100,000 – $124,999<br>6=$125,000 – $149,999<br>7=$150,000 or greater
Household size	Including yourself, how many people currently live in your household?<br><br>**[page break]**	[Drop-down menu display with options from 1 to 20]
	**F: CLOSURE**	

© 2022 Musicus AA et al. *JAMA Network Open.*

Construct	Item	Response scale<br>(all force response)
Debrief	Thank you for taking part in this research study. There was some information about the study that we did not share with you at the beginning of your participation so that you would react honestly. We would now like to take this opportunity to fully inform you of the nature of this research and explain a bit more about the study in which you just participated.<br><br>At the beginning of the survey we indicated that you would either receive the drink you selected or additional compensation worth $5. **<u>In actuality, you and all other study participants will receive additional compensation worth $5 for your participation in this study in addition to the amount agreed upon when you entered this survey.</u>** We stated that you might receive the drink you selected because we wanted you to behave as you normally would when shopping online and choose a drink you would actually want to purchase for your child. This study was conducted by researchers at the Harvard T.H. Chan School of Public Health who are interested in the effects of front-of-package labeling elements (claims, imagery, disclosures, and warnings) on fruit-flavored drinks. We will use this study to determine how effective different types of front-of-package labels are at informing consumers about how much added sugar is in their drinks, and whether the labels change which drink people choose.<br><br>Please note that this additional $5 incentive will not be delivered immediately, but may take up to 3-4 weeks to process. In addition, responses will be reviewed for quality purposes and compensation and will only be given to those who **honestly** completed the survey. However, if you have any questions or concerns regarding your response or about your participation, please contact the investigator, Dr. Eric Rimm. He can be reached via email at erimm@hsph.harvard.edu. If you would like a copy of this debriefing form, please save this page to your computer.<br><br>Please choose one of the options below.<br><br>**[page break]**	1=I agree to have my data included in this study.<br>2=I no longer wish to participate and/or I would like my data removed from the study. (Your data will be removed from the study entirely, except for the consent and debriefing form).
Confirm data removal	**[only display if Debrief=2]**<br><br>Please confirm that you no longer wish to participate and/or would like your data removed from the study.<br><br>**[page break]**	1=Confirm<br>2=I would like my data included in the study.

© 2022 Musicus AA et al. *JAMA Network Open.*

Construct	Item	Response scale<br>(all force response)
Completion code	Your code for completion of this survey: ${e://Field/GeneratedNumber}<br><br>Please copy this number. If you should need to contact the researchers regarding your response, **please include this number** so they can look up your response. Please hit the '→' button in order to submit your response.<br><br>**[page break]**	
End	We thank you for your time spent taking this survey. Your response has been recorded.	

© 2022 Musicus AA et al. *JAMA Network Open.*

eAppendix 2. Image Information

**BEVERAGE CHOICE TASK**							
**Brand,* Flavor**	**Beverage Category**	**Serving Size**	**Calories/Serving**	**Added Sugar (g)/Serving**	**Total Sugar (g)/Serving**	**Percent Juice Content**	**Contains Non-Nutritive Sweeteners**
Brand A, Fruit Punch	High-added-sugar fruit drink	6 oz pouch	60	11	13	10%	No
Brand B, Pink Lemonade	High-added-sugar fruit drink	6.75 oz pouch	90	24	24	12%	No
Brand C, Cranberry-Raspberry	High-added-sugar fruit drink	10 oz bottle	130	28.75	32.5	15%	No
Brand D, Grape	High-added-sugar fruit drink	10 oz bottle	140	29	32	7%	Yes: sucralose
Brand E, Orange Soda	Soda (high-added-sugar)	10 oz bottle	135	36	36	0%	No
Brand F, Cola	Soda (high-added-sugar)	10 oz bottle	120	32.5	32.5	N/A	No
Brand G, Grape	Low-added-sugar fruit drink	6 oz box	40	8	10	10%	Yes: sucralose, acesulfame potassium
Brand H, Fruit Punch	No-added-sugar fruit drink	6 oz box	35	0	8	38%	No
Brand I, Apple	100% juice	6.75 oz box	100	0	22	100%	No
Brand J, Orange	100% juice	8 oz carton	110	0	22	100%	No
Brand K, Milk	Milk	8 oz box	150	0	12	N/A	No
Brand L, Water	Water	8 oz bottle	0	0	0	N/A	No
**BEVERAGE PERCEPTION/KNOWLEDGE TASK**							
**Brand,* Flavor**	**Beverage Category**	**Serving Size**	**Calories/Serving**	**Added Sugar (g)/Serving**	**Total Sugar (g)/Serving**	**Percent Juice Content**	**Contains Non-Nutritive Sweeteners**
Brand C, Cranberry-Apple	High-added-sugar fruit drink	10 oz bottle	130	28.75	32.5	15%	No
Brand A, Strawberry Kiwi	High-added-sugar fruit drink	6 oz pouch	60	12	13	10%	No
Brand G, Orange	Low-added-sugar fruit drink	6 oz box	40	8	10	10%	Yes: sucralose, acesulfame potassium
Brand H, Strawberry Peach	No-added-sugar fruit drink	6 oz box	35	0	5	40%	No

*Brand names replaced with letters. Images used in survey were sized at 600px width.

eAppendix 3. Preregistration

AS PREDICTED

Wharton CREDIBILITY LAB UNIVERSITY of PENNSYLVANIA

Front-of-package claims, imagery, disclosures, and warnings: Online RCT (#61291)

Created: 03/18/2021 02:05 PM (PT)

Public: 04/23/2022 01:44 PM (PT)

Author(s)

Aviva Musicus (Harvard T.H. Chan School of Public Health) - aam231@mail.harvard.edu Christina Roberto (University of Pennsylvania) - croberto@pennmedicine.upenn.edu Alyssa Moran (Johns Hopkins Bloomberg School of Public Health) - amoran10@jhu.edu Sarah Sorscher (Center for Science in the Public Interest) - ssorscher@cspinet.org Eric Rimm (Harvard T.H. Chan School of Public Health) - erimm@hsph.harvard.edu

1. **Have any data been collected for this study already?**

No, no data have been collected for this study yet.

2. **What's the main question being asked or hypothesis being tested in this study?**

Study aim: Test the independent and combined effects of front-of-package (FOP) claims, imagery, disclosures, and added sugar warning labels on parents' choices and perceptions of beverages for their children using an online randomized controlled design. Hypothesis: Warning labels with or without added sugar disclosures in teaspoons will reduce purchases of labeled beverages compared to the other conditions. A FOP % juice disclosure will increase consumer knowledge about % juice in the product, and a FOP teaspoons of added sugar warning label will increase consumer knowledge about the amount of added sugar in the product.

3. **Describe the key dependent variable(s) specifying how they will be measured.**

The primary outcomes will be total calories and grams of added sugar chosen in the online store (averaged by condition). We will also examine the percentage of people in each condition that purchased a drink high in added sugars (>20% DV) for their child, and the percentages that chose each category of drink (fruit drinks, 100% juice, etc).

4. **How many and which conditions will participants be assigned to?**

Caregivers will be randomized to see drinks in our online store and survey with one of the following seven label conditions. The first condition (claim & imagery, control) will represent the status quo and contain a 100% Vitamin C claim and fruit imagery. Conditions 2-4 will involve the removal of package elements for fruit drinks high in added sugars (>10g/serving, 20% DV): Condition 2 (imagery only) will have fruit imagery but no 100% Vitamin C claim on high-added-sugar fruit drinks; Condition 3 (claim only) will have a 100% Vitamin C claim but no fruit imagery on high-added-sugar fruit drinks; and Condition 4 (no claim or imagery) will feature no imagery or claims on high-added-sugar fruit drinks. Conditions 5-7 will involve the addition of package elements. Condition 5 (imagery, claim, & text % fruit juice disclosure) will show a text description of the percent fruit juice in each fruit drink/juice, to see if it changes the effects of the claim and imagery. The sixth (imagery, claim, & added sugar warning) and seventh conditions (imagery, claim, & added sugar warning w/teaspoons of sugar) will test the effects of added sugar warnings.

5. **Specify exactly which analyses you will conduct to examine the main question/hypothesis.**

Linear regression will be used to examine mean total calories and grams of added sugar purchased between our experimental groups. Logistic regression will be used to compare proportions in each condition that chose different beverage types. Because of the randomized design, the only covariates we will control for will be purchasing frequency (for secondary outcomes), and those that may by chance significantly differ among the groups. A Bonferroni-Holm correction will be used to control for multiple comparisons.

6. **Describe exactly how outliers will be defined and handled, and your precise rule(s) for excluding observations.**

We will exclude observations based on the following criteria:

- Participants who complete the survey in less than 1/3 of the median completion time
- Observations from duplicate IP addresses (keep the first observation that is unique)
- Participants who answer data integrity check incorrectly
- Participants that do not meet the inclusion criteria (primary caregiver of a child 0-5 years old; >=18 years old; U.S. resident)

7. How many observations will be collected or what will determine sample size? No need to justify decision, but be precise about exactly how the number will be determined.

We determined that a sample of 4,900 participants (700 per group) would provide at least 80% power to detect an 18-kcal difference between each group, assuming an alpha of 0.05. This estimate is based on effect sizes found in our previous labeling research. It is typical to exclude a small number of participants in online studies (e.g., for failing data integrity check questions), so we will recruit 5,000 participants.

8. Anything else you would like to pre-register? (e.g., secondary analyses, variables collected for exploratory purposes, unusual analyses planned?)

Below we detail our planned secondary analyses:

- In the beverage perception task, results will be examined individually by drink and averaged for the two low-added-sugar fruit drinks and for the two high-added sugar fruit drinks-we will examine differences across conditions: Likelihood to buy for child, how healthy for child, how appealing to child, disease risk perceptions for child (gain excess weight, type 2 diabetes, healthier life), Perceived Message Effectiveness for beverage package, % fruit juice in product, quantity of added sugars in product (categorical and continuous)
- Label recall
- Demographics: age, gender, ethnicity/race, BMI, # and age of children, diet-related diseases, education, SNAP, WIC, income, frequency of child fruit drink consumption
- Exploratory analyses will include stratification/effect measure modification of main effects by SNAP/WIC status, race/ethnicity, household income, and frequency of child fruit drink consumption.

Pre-registration Note: Although the pre-analysis plan specified using the Bonferroni-Holm method to correct for multiple comparisons, this type of multiple test adjustment has been criticized for increasing the likelihood of type II errors, obscuring potentially important differences.[1] Based on recommendations in the scientific literature,[1] we have instead described the tests we performed and presented 95% confidence intervals with uncorrected p-values.

1. Perneger TV. What's wrong with Bonferroni adjustments. *BMJ.* 1998;316(7139):1236-1238.

eTable 1. Effect of Front-of-Package Modifications on Beverage Choice, Mean Values (SE)

	**Control (claim & image) (n=714)**	**No Claim (Imagery only) (n=717)**	**No Imagery (Claim only) (n=710)**	**No Claim or Imagery (n=717)**	**Percent Juice Disclosure (n=708)**	**Warning (n=729)**	**Warning & Teaspoon Disclosure (n=710)**
**Beverage Choice**	**Mean (SE)**						
Chose high-added-sugar beverage, %	41.0 (1.8)	38.8 (1.8)	38.7 (1.8)	**33.5 (1.8)**	39.3 (1.8)	**35.5 (1.8)**	**34.7 (1.8)**
Chose high-added-sugar fruit drink, %	33.6 (1.8)	31.2 (1.7)	29.9 (1.7)	**25.7 (1.6)**	31.9 (1.8)	**27.6 (1.7)**	**27.5 (1.7)**
Chose no-added-sugar fruit drink, %	16.8 (1.4)	**21.6 (1.5)**	**21.6 (1.5)**	**21.1 (1.5)**	17.1 (1.4)	19.5 (1.5)	**23.9 (1.6)**
Chose 100% juice	24.7 (1.6)	22.7 (1.6)	23.1 (1.6)	27.2 (1.7)	27.8 (1.7)	26.1 (1.6)	22.5 (1.6)
Chose low-added-sugar fruit drink, %	6.0 (0.9)	5.9 (0.9)	5.2 (0.8)	7.0 (1.0)	5.2 (0.8)	7.5 (1.0)	4.9 (0.8)
Chose soda, %	7.4 (1.0)	7.5 (1.0)	8.9 (1.1)	7.8 (1.0)	7.3 (1.0)	8 (1.0)	7.2 (1.0)
Chose milk, %	5.9 (0.9)	5.9 (0.9)	5.6 (0.9)	6.7 (0.9)	4.7 (0.8)	5.6 (0.9)	6.5 (0.9)
Chose water, %	5.6 (0.9)	5.2 (0.8)	5.8 (0.9)	4.6 (0.8)	5.9 (0.9)	5.8 (0.9)	7.5 (1.0)
Calories in chosen beverage, kcal	81.9 (1.6)	80.6 (1.6)	79.9 (1.6)	81.2 (1.6)	81.2 (1.6)	80.1 (1.6)	**76.5 (1.6)**
Added sugar in chosen beverage, g	9.4 (0.5)	9.3 (0.5)	9.2 (0.5)	8.1 (0.4)	8.9 (0.4)	8.6 (0.4)	**8.0 (0.4)**
Total sugar in chosen beverage, g	17.7 (0.4)	17.5 (0.4)	17.4 (0.4)	17.3 (0.4)	17.7 (0.4)	17.3 (0.4)	**16.3 (0.4)**
Among high-added-sugar fruit drink selections (n=1,482)							
Chose high-added-sugar fruit drink bottle, %	30.4 (0.3)	33.0 (0.3)	29.3 (0.3)	32.1 (0.3)	30.1 (0.3)	31.8 (0.3)	28.2 (0.3)
Chose high-added-sugar fruit drink pouch, %	69.6 (0.3)	67.0 (0.3)	70.8 (0.3)	67.9 (0.3)	69.9 (0.3)	68.2 (0.3)	71.8 (0.3)

Bold values indicate significant difference from control, p<0.05

© 2022 Musicus AA et al. *JAMA Network Open.*

eFigure 1. Interaction Between Special Supplemental Nutrition Program for Women, Infants, and Children Status and No Claim or Imagery Label Condition on Mean Calories and Added Sugar

Error bars show standard errors.

A. Calories, No Claim or Imagery vs. Control

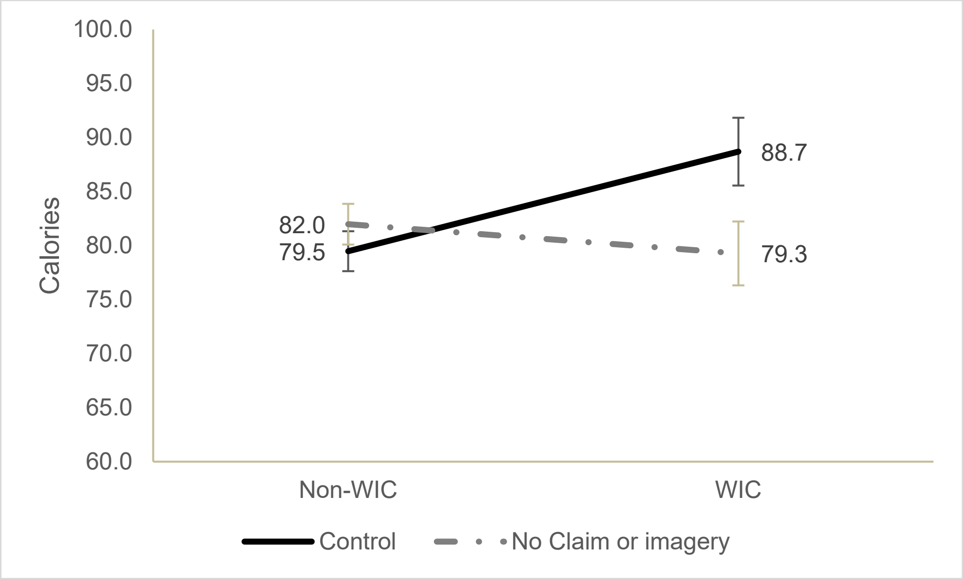

B. Added sugar (grams), No Claim or Imagery vs. Control

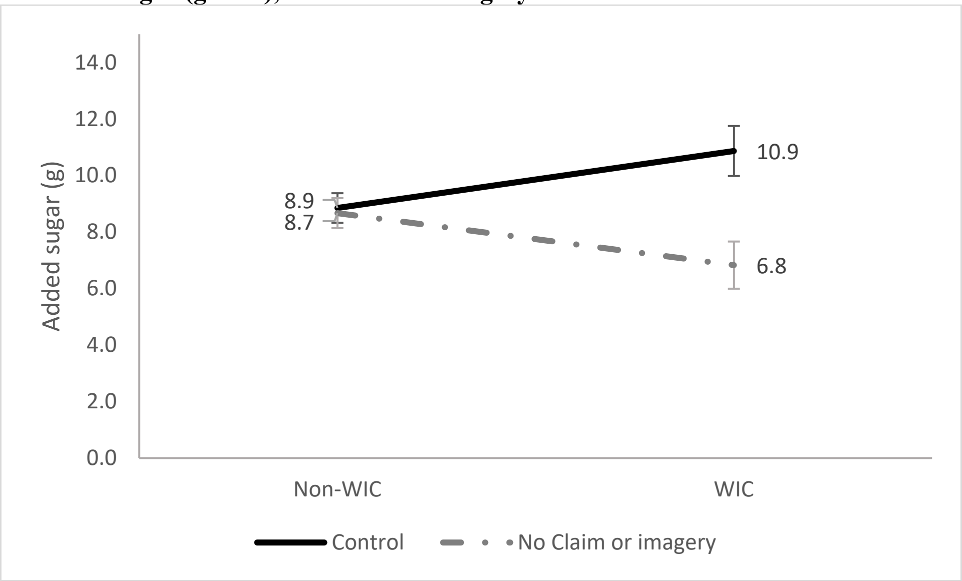

© 2022 Musicus AA et al. *JAMA Network Open.*

eFigure 2. Interaction Between Hispanic Ethnicity and Label Conditions in Effect on Mean Calories
Error bars show standard errors.
A. Calories, No Claim vs. Control

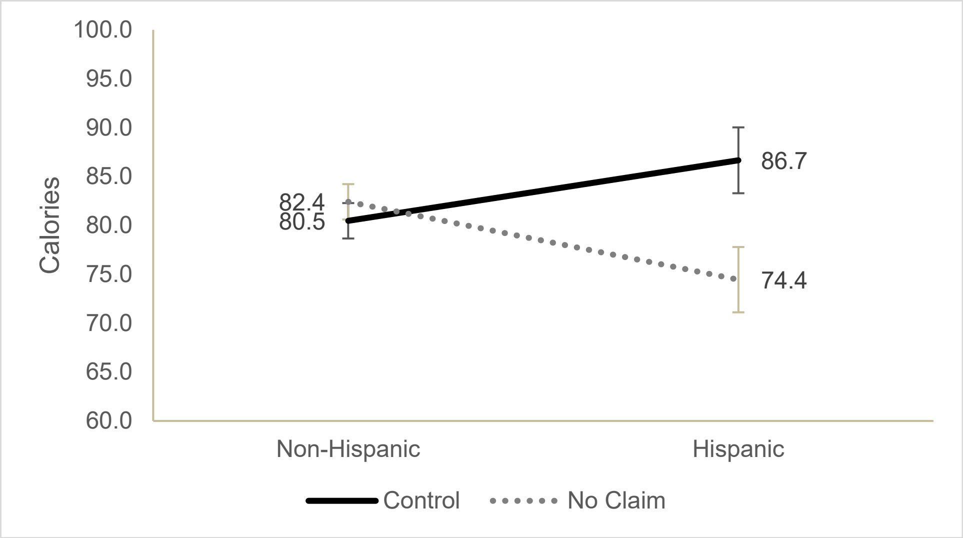

B. Calories, No Imagery vs. Control

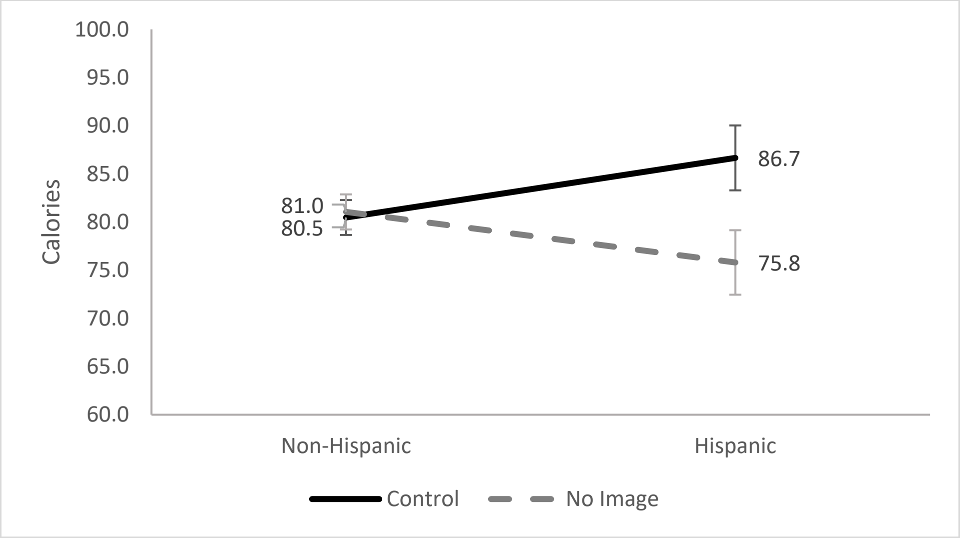

C. Calories, Warning vs. Control

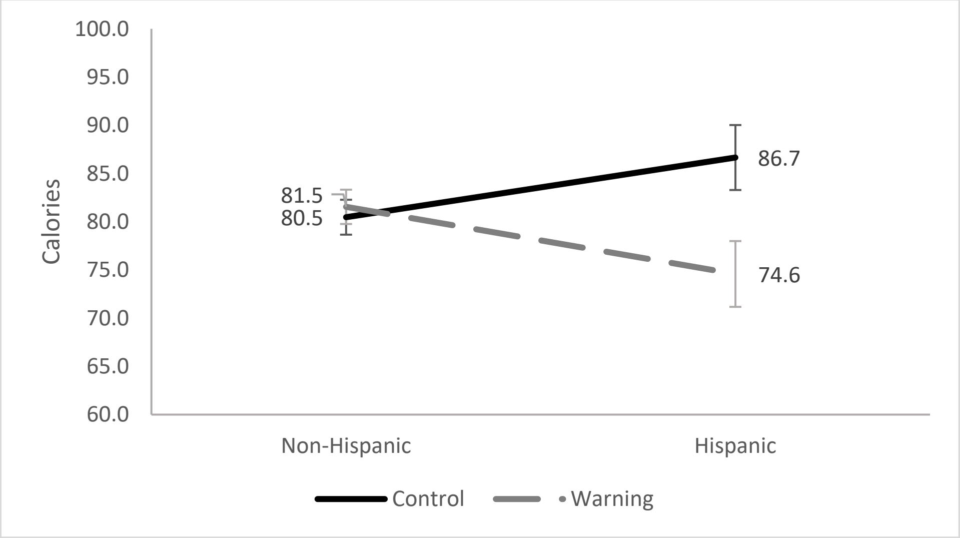

© 2022 Musicus AA et al. *JAMA Network Open.*

eTable 2. Individual Fruit Drink Knowledge and Perceptions

	Control (claim + imagery) (n=714)	No Claim (n=717)	No Imagery (n=710)	No Claim or Imagery (n=717)	Percent Juice Disclosure (n=708)	Warning (n=729)	Warning & Teaspoon Disclosure (n=710)
**High-added-sugar fruit drink, cranberry-apple flavor, Knowledge & Perceptions (high-added-sugar, 28.75g, 7tsp; 15% juice)**							
Percent juice estimate (correct = 15%), mean (SE)	50.5 (1.1)	48.7 (1.1)	48.8 (1.1)	51 (1.1)	**23.4 (1.1)**	**41.6 (1.1)**	**39.8 (1.1)**
Thought fruit drink was 100% juice, %	10 (1.1)	7.4 (1)	10.5 (1.1)	9 (1.1)	**1.5 (0.5)**	7.2 (1)	**6.9 (1)**
Added sugar estimate (correct = 7 tsp), mean tsp (SE)	5 (0.3)	5.2 (0.3)	5.5 (0.3)	4.4 (0.3)	5.8 (0.3)	**7.7 (0.3)**	**7.2 (0.3)**
Added sugar estimate, mean (SE) [None=1, A little=2, Some=3, A lot=4]	2.6 (0)	2.6 (0)	2.6 (0)	**2.5 (0)**	**2.8 (0)**	**3.2 (0)**	**3.3 (0)**
Likelihood to buy for child, mean (SE) [1-7]	3.9 (0.1)	4.1 (0.1)	3.8 (0.1)	3.8 (0.1)	3.8 (0.1)	**3.6 (0.1)**	**3.6 (0.1)**
Appeal to child, mean (SE) [1-7]	4.5 (0.1)	4.4 (0.1)	**3.9 (0.1)**	**3.7 (0.1)**	4.4 (0.1)	**4.2 (0.1)**	4.3 (0.1)
Health perception index, mean (SE) [4-28]	17.9 (0.2)	17.9 (0.2)	17.9 (0.2)	18.2 (0.2)	**17.1 (0.2)**	**15.3 (0.2)**	**15.1 (0.2)**
Perceived Message Effectiveness, mean (SE) [1-5]	2.6 (0)	2.7 (0)	2.7 (0)	2.5 (0)	**2.9 (0)**	**3.4 (0)**	**3.4 (0)**
**High-added-sugar fruit drink, strawberry-kiwi flavor, Knowledge & Perceptions (high-added-sugar, 12g, 3tsp; 10% juice)**							
Percent juice estimate (correct = 10%), mean (SE)	41.8 (1.1)	**38.1 (1.1)**	39.2 (1.1)	39.1 (1.1)	**18.7 (1.1)**	**36 (1.1)**	**36.5 (1.1)**
Thought fruit drink was 100% juice, % (SE)	6.4 (0.9)	4.8 (0.8)	6 (0.9)	4.5 (0.8)	**1.6 (0.5)**	4.2 (0.7)	5.5 (0.8)
Added sugar estimate (correct = 3 tsp), mean tsp (SE)	5 (0.3)	5.4 (0.3)	**6.2 (0.3)**	5.3 (0.3)	6 (0.3)	**7.5 (0.3)**	5.6 (0.3)
Added sugar estimate, mean (SE) [None=1, A little=2, Some=3, A lot=4]	2.9 (0)	2.9 (0)	2.9 (0)	2.9 (0)	3 (0)	**3.3 (0)**	**3.2 (0)**
Likelihood to buy for child, mean (SE) [1-7]	4.8 (0.1)	4.6 (0.1)	4.6 (0.1)	**4.6 (0.1)**	4.6 (0.1)	**4.3 (0.1)**	**4.5 (0.1)**
Appeal to child, mean (SE) [1-7]	5.5 (0.1)	5.5 (0.1)	**4.7 (0.1)**	**4.7 (0.1)**	**5.3 (0.1)**	**5.2 (0.1)**	**5.3 (0.1)**
Health perception index, mean (SE) [4-28]	16 (0.2)	**15.4 (0.2)**	15.6 (0.2)	**15.4 (0.2)**	**15.2 (0.2)**	**13.9 (0.2)**	**14.4 (0.2)**
Perceived Message Effectiveness, mean (SE) [1-5]	2.8 (0)	2.9 (0)	2.8 (0)	2.8 (0)	**3 (0)**	**3.5 (0)**	**3.3 (0)**
**Low-added-sugar fruit drink, orange flavor, Knowledge & Perceptions (low-added-sugar, 8g; 10% juice)**							
Percent juice estimate (correct = 10%), mean (SE)	38.9 (1.1)	38 (1.1)	37.4 (1.1)	38.5 (1.1)	**18.4 (1.1)**	37.8 (1.1)	37.6 (1.1)
Thought fruit drink was 100% juice, % (SE)	6.6 (0.9)	6.5 (0.9)	5.7 (0.9)	4.3 (0.8)	**0.7 (0.3)**	4.7 (0.8)	5.8 (0.8)
Added sugar estimate (correct = 2 tsp), mean tsp (SE)	5.9 (0.4)	6 (0.4)	**7 (0.4)**	6 (0.4)	6.6 (0.4)	6.4 (0.4)	6 (0.4)
Added sugar estimate, mean (SE) [None=1, A little=2, Some=3, A lot=4]	3.1 (0)	3.1 (0)	3.1 (0)	3.1 (0)	3.1 (0)	3 (0)	**3 (0)**
Likelihood to buy for child, mean (SE) [1-7]	4 (0.1)	3.9 (0.1)	3.9 (0.1)	4 (0.1)	3.8 (0.1)	4 (0.1)	4 (0.1)
Appeal to child, mean (SE) [1-7]	5.1 (0.1)	5.1 (0.1)	5.2 (0.1)	**5.4 (0.1)**	5 (0.1)	5.2 (0.1)	5.1 (0.1)
Health perception index, mean (SE) [4-28]	14.7 (0.2)	14.5 (0.2)	14.7 (0.2)	14.8 (0.2)	**14.2 (0.2)**	14.4 (0.2)	14.5 (0.2)
Perceived Message Effectiveness, mean (SE) [1-5]	3.1 (0)	3.1 (0)	3.1 (0)	2.9 (0)	**3.2 (0)**	3 (0)	3 (0)
**No-added-sugar fruit drink, strawberry peach flavor, Knowledge & Perceptions (no-added-sugar, 0g; 38% juice)**							
Percent juice estimate (correct = 38%), mean (SE)	51.2 (1.2)	51.3 (1.2)	52.9 (1.2)	53.6 (1.2)	**38.1 (1.2)**	49.5 (1.2)	48.2 (1.2)
Thought fruit drink was 100% juice, % (SE)	9.9 (1.1)	10.5 (1.1)	12 (1.2)	11.6 (1.2)	**1.4 (0.5)**	10 (1.1)	9.1 (1.1)
Added sugar estimate (correct = 0 tsp), mean tsp (SE)	3.8 (0.3)	3.8 (0.3)	4.1 (0.3)	3.4 (0.3)	4.2 (0.3)	4.2 (0.3)	4.1 (0.3)
Added sugar estimate, mean (SE) [None=1, A little=2, Some=3, A lot=4]	2.4 (0)	2.4 (0)	2.4 (0)	2.3 (0)	**2.5 (0)**	2.4 (0)	2.4 (0)
Likelihood to buy for child, mean (SE) [1-7]	4.5 (0.1)	4.6 (0.1)	4.6 (0.1)	**4.7 (0.1)**	4.6 (0.1)	4.6 (0.1)	4.6 (0.1)
Appeal to child, mean (SE) [1-7]	4.9 (0.1)	4.9 (0.1)	5.1 (0.1)	**5.2 (0.1)**	5 (0.1)	4.9 (0.1)	5 (0.1)
Health perception index, mean (SE) [4-28]	18.7 (0.2)	18.7 (0.2)	19.1 (0.2)	**19.2 (0.2)**	18.5 (0.2)	18.5 (0.2)	**18.1 (0.2)**
Perceived Message Effectiveness, mean (SE) [1-5]	2.5 (0)	2.5 (0)	2.5 (0)	2.4 (0)	**2.6 (0)**	2.6 (0)	2.6 (0)

© 2022 Musicus AA et al. *JAMA Network Open.*

Bold values indicate significant difference from control, $p<0.05$

© 2022 Musicus AA et al. *JAMA Network Open.*
