# Supplementary material for: Effect of Front-of-Package Information, Fruit Imagery, and High–Added Sugar Warning Labels on Parent Beverage Choices for Children: A Randomized Clinical Trial
Source: JAMA Netw Open. 2022 Oct 13;5(10):e2236384. doi: 10.1001/jamanetworkopen.2022.36384 (PMC9561948; doi:10.1001/jamanetworkopen.2022.36384)
Supplement: Supplement 3. — Data Sharing Statement [file jamanetwopen-e2236384-s003.pdf]

Data Sharing Statement

Musicus AA, Roberto CA, Moran AJ, Sorscher S, Greenthal E, Rimm EB. Effect of front-of-package information, fruit imagery, and high-added sugar warning labels on parent beverage choices for children. *JAMA Netw Open*. 2022;5(10):e2236384. doi:10.1001/jamanetworkopen.2022.36384

Data
Data available: No

© 2022 Musicus AA et al. *JAMA Network Open*.
